# [BMIM][X] Ionic Liquids Supported on a Pillared-Layered Metal–Organic Framework: Synthesis, Characterization, and Adsorption Properties

**DOI:** 10.3390/molecules29153644

**Published:** 2024-08-01

**Authors:** Yaiza Martín-García, Jesús Tapiador, Gisela Orcajo, Juan Ayala, Ana B. Lago

**Affiliations:** 1 Laboratorio de Materiales para Análisis Químico (MAT4LL), Departamento de Química, Unidad Departamental de Química Inorgánica, Universidad de La Laguna (ULL), 38206 San Cristóbal de La Laguna, Spain; alu0100823448@ull.edu.es; 2 Department of Chemical, Energy and Mechanical Technology, Rey Juan Carlos University, Calle Tulipán s/n, 28933 Móstoles, Spain; jesus.tapiador@urjc.es (J.T.); gisela.orcajo@urjc.es (G.O.); 3 Laboratorio de Materiales para Análisis Químico (MAT4LL), Departamento de Química, Unidad Departamental de Química Analítica, Universidad de La Laguna (ULL), 38206 San Cristóbal de la Laguna, Spain; jayala@ull.edu.es

**Keywords:** metal–organic framework, pillared-layered frameworks, composite compounds, ionic liquids

## Abstract

Combining ionic liquids (ILs) and metal–organic frameworks (MOFs) can be an intriguing opportunity to develop advanced materials with different adsorption capabilities for environmental applications. This study reports the preparation and characterization of a 3D pillared-layered compound, namely, [Zn_2_(tz)_2_(bdc)] (CIM91), formed by 1,2,4-triazole (Htz) and 1,4-benzenedicarboxylic acid (H_2_bdc) ligands. Then, various loadings of the water-stable and hydrophobic IL, 1-n-butyl-3-methylimidazolium hexafluorophosphate ([BMIM][PF_6_]), and the water-soluble 1-n-butyl-3-methylimidazolium chloride ([BMIM][Cl]) were incorporated into CIM91. Detailed characterization by X-ray powder diffraction (XRD), FT-IR spectra, scanning electron microscopy (SEM), Energy dispersive X-ray (EDX) analysis, N_2_ adsorption measurements, and thermogravimetric analysis confirmed the formation of [BMIM][X]/CIM91 composites and the structural stability of the MOF after the incorporation of the ionic liquids. CO_2_ adsorption–desorption analysis was experimentally carried out for all the materials at 298 K and 318 K, demonstrating a great enhancement in the CO_2_ adsorption properties of the sole MOF CIM91, particularly by including [BMIM][PF_6_] species in its structure with a double isosteric heat of CO_2_ adsorption. The composites were also tested for the adsorption of methylene blue (MB) dye. The results indicate that the incorporation of [BMIM][X] into CIM91 can substantially modify the adsorption properties of the MOF. The influence of the nature of the [BMIM][X] anions on these properties has also been analyzed.

## 1. Introduction

The post-synthesis modification of a porous compound offers the opportunity to design a composite material with task-specific properties desirable for any targeted application. The mixture of two different constituents combined at a molecular level is known as a hybrid material. In this regard, hybrid composites that can be synthesized by combining solid porous materials with ILs with different physicochemical properties provide various advantages [1].

Ionic liquid compounds are organic salts with low melting points (usually below 100 °C). All the ILs share several noteworthy properties—low vapor pressure, wide liquidus range, relatively high viscosity, and a remarkable capability of dissolving various organic or inorganic materials. Besides, they can be tuned to improve one or several of their properties by combining appropriate ions or functionalizing their organic moieties. The thermophysical properties of ILs can be modified by choosing the anion and substituents of the cation. In this sense, properties such as thermal stability, density, or heat capacity increase with increasing anion sizes [2].

Metal–organic frameworks (MOFs) are coordination networks containing potential voids [3]. The huge advantage of MOFs compared to other porous compounds is the ability to tune the individual building blocks and therefore, the properties of the related porous hybrid materials on the molecular level [4]. Pillared-layer metal–organic frameworks are a subset of MOFs with easy tunability [5] and composed of two sets of organic linkers, where one ligand links the metal clusters into layers and the other donor ligand pillars the layers into a 3D framework. In particular, the series based on Zn(II)-triazolate layers has been highlighted for its ability to trap CO_2_ molecules inside [6].

ILs are often used as a green solvent or a structure-directing agent in the synthesis of new hybrid materials with MOF compounds. This synthetic approach is often used when new compounds or modified structures are desirable. However, post-synthesis modification strategies are used when no structural changes are pursued in the MOF compounds or to provide a more straightforward methodology synthetic route.

This study focuses on the well-known imidazolium-based salts and, in particular, seeks to understand how the anion can affect the adsorption properties of the final composite compounds.

We have previously synthesized and characterized the terephthalate pillared-layer Zn-triazolate metal–organic framework (CIM-81) and used it as a sorbent in a dispersive micro-solid phase extraction method for a group of different personal care products in complex wastewater, demonstrating better analytical performance features than other MOFs that had already been used with success, such as HKUST-1, MIL-53(Al), and UiO-66 [7].

We also analyzed the interactions between a series of parabens and the Zn-layered pillared MOFs, CIM-81, and their amino derivatives CIM-82 and CIM-83. The possible interactions established between these MOFs and the studied analytes, as a function of the nature of the functionalization of the organic ligand in the MOF, have also been studied [8].

Potentially, the self-assembly of the same inorganic and organic building blocks under different conditions can give rise to different MOFs with the same chemical composition but different network superstructures, which are known as supramolecular isomers [9], specifically termed pseudo-polymorphous compounds, if the difference is due to the guest molecules occupied in the pore [10,11].

The properties of the MOF can be tuned by varying the structure type, organic functionality, or geometry of the metal-containing unit, together with the size of the pores or the nanocrystals. For these materials, ILs can be used as modifying agents providing further flexibility because of their tunability by changing cations and anions to meet the needs of any specific application [12].

The early applications of MOFs focused on gas storage, separation, and toxic gas adsorption [3], but recently, the need for CO_2_ emission reduction has boosted their interest due to the durability and CO_2_ capture data based on the industrial tests of some MOFs [3,13].

The immobilization of ILs in MOFs offers a broad potential to form composite materials showing superior performances in many applications [1,14], mostly focusing on gas applications, such as catalysis [15,16,17], adsorption [18], and membrane-based separation [19]. IL/MOF composites can also be potentially used to remove bigger molecules. Some dyes, such as methylene blue (MB), are resistant to common oxidizing agents or heat treatments and adsorption-based separation offers the most suitable way to remove these molecules [20]. Composites can be tuned to selectively remove contaminants with the existence of an almost-limitless number of IL-MOF combinations offering a high degree of flexibility [21,22].

In this study, a water-stable and hydrophobic IL [23], 1-n-butyl-3-methylimidazolium hexafluorophosphate ([BMIM][PF_6_]), and the water-soluble 1-n-butyl-3-methylimidazolium chloride ([BMIM][Cl]) were incorporated into the [Zn_2_(tz)_2_(bdc)] (Htz = 1,2,4-triazole and H_2_bdc = 1,4-benzenedicarboxylic acid), CIM91, pillared-layer metal–organic framework. We then investigated the adsorption capabilities of these composites to remove CO_2_ and the methylene blue (MB) dye.

## 2. Results and Discussion

### 2.1. Synthesis and Characterization

CIM91 was prepared in good yield by the solvothermal self-assembly of triazolate and carboxylate ligands and Zn(II) nitrate salt in the ratio 1:0.5:1 using DMF as a solvent. The temperature was increased concerning the previously published syntheses [24] to optimize the synthesis and scale it for large material production.

The diffraction patterns obtained for the compound reveal that the materials are consistent with the simulated one from the single-crystal structure, indicating the phase purity of the as-synthesized sample. (Figures S1–S3)

**Figure 1:**
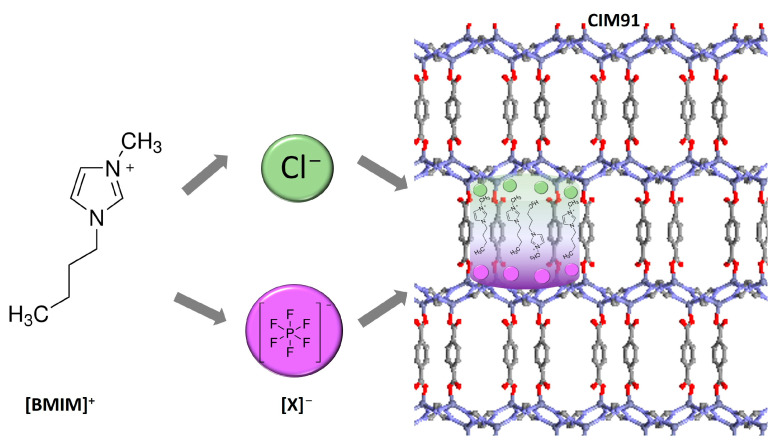
Representation of the [BMIM][Cl] and [BMIM][PF_6_] ILs and the 3D structure of CIM91.

The wet impregnation method was used to prepare the different IL/CIM91 composites (Figure 1) as this method was more beneficial for obtaining higher IL loadings. The IL-incorporated CIM91 materials were prepared by adding various amounts of ILs in the synthetic procedure, 40, 20, and 10 wt% in [BMIM][PF_6_]/CIM91 and 20, 10, and 5 wt% in [BMIM][Cl]/CIM91, according to IL weight percent. Different solvents were considered for impregnation: acetone and a mixture of methanol–tetrahydrofuran. Both the solvents can readily dissolve [BMIM][Cl] and [BMIM][PF_6_]. The as-synthesized CIM91 compound was also used to guarantee MOF stability and to obtain the composite material through an exchange solvent process between the DMF solvent molecules and [BMIM][X] compounds. Nevertheless, when the as-synthesized CIM91 compound was used with [BMIM][PF_6_], any or lower IL loadings were observed, and a mixture of DMF and IL was obtained when [BMIM][Cl] was used. So, suitable impregnation could only be achieved when activated CIM91 was used.

The resulting final mixtures were stirred at room temperature for 3 h followed by solvent evaporation. The final IL/CIM91 composites were dried at 90 °C with a vacuum to remove the solvents and to obtain the final white composite powders.

The [BMIM][PF_6_]/CIM91 composite exhibits no alterations in its crystal structure following the introduction of IL, indicating that the crystal structure of CIM91 remained intact during IL impregnation, consistent also with the SEM images (Figures S5 and S6). As a result, it can be concluded that the structures did not show any deformation or loss of crystallinity after impregnation. However, the diffraction pattern obtained by the composite formed by [BMIM][Cl] and CIM91 shows a degradation of the original MOF structure as the ionic liquid loading increases. The appearance of new peaks is observed above a load of 20 wt% IL and at higher loadings (>40 wt%), the sample lost the original crystallinity (Figures S2 and S3). This is the reason why we limit the [BMIM][Cl] loading to 20 wt%.

The compounds were air-stable, and their structures were almost undamaged after being soaked in acetone (Figure S4).

**Figure 2:**
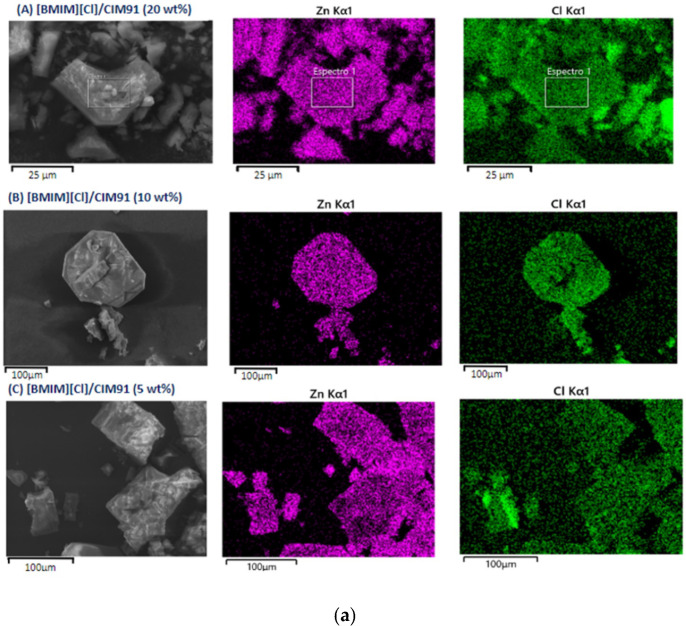

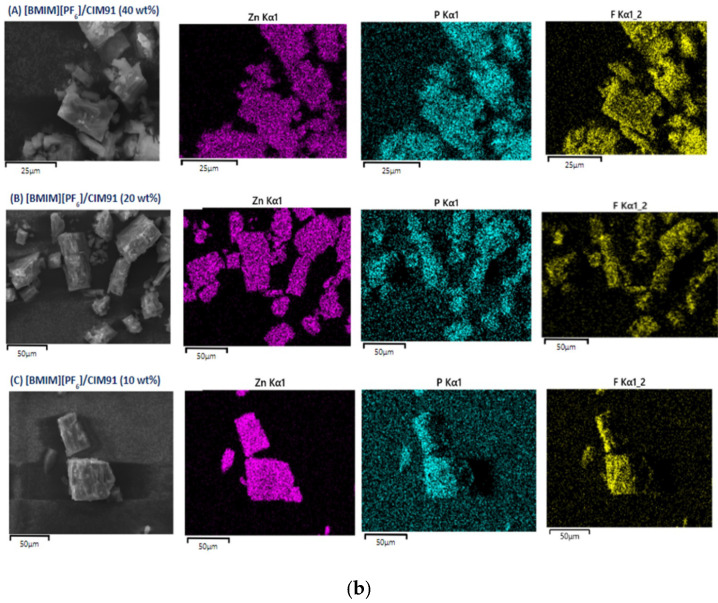
SEM images of composite crystalline material: (**a**) [BMIM][Cl]/CIM91; (**b**) [BMIM][PF_6_]/CIM91. Energy dispersive X-ray (EDX) mapping analysis of composites: Zn and Cl atoms are shown in (**a**) and Zn, P, and F atoms in (**b**).

The photomicrographs of the different [BMIM][PF_6_]/CIM91 and [BMIM][Cl]/CIM91 samples were obtained by SEM, and they are provided in Figure 2. The size and shape of the particles are almost identical to those of CIM91, showing that the crystal structure is preserved during the incorporation of both ILs into CIM91. The EDX analysis is used to study the Zn:P and Zn:Cl atomic relations in the [BMIM][PF_6_]/CIM91 and [BMIM][Cl]/CIM91 compounds (Table S1). The composites prepared showed a molar ratio of Zn/Cl in agreement with the theoretical values, whereas the amount of PF_6_^-^ observed is above the expected value in [BMIM][PF_6_]/CIM91 (10 wt%). These results agree with the SEM-EDX mapping images shown in Figure 2. Furthermore, these images prove that CIM91 and [BMIM][X] (X = PF_6_ and Cl) are homogeneously dispersed. The elemental analysis confirms the corresponding IL loadings of the [BMIM][PF_6_]/CIM91 and [BMIM][Cl]/CIM91 samples (Table S2).

N_2_ adsorption measurements were performed for the [BMIM][PF_6_]/CIM91 and [BMIM][Cl]/CIM91 samples at 77 K between 0 and 1 bar to determine the BET surface areas. The adsorption isotherms and the resulting BET surface areas are given in Figures S7 and S8 and Table S3, respectively. The N_2_ adsorption at 77 K yielded a type I isotherm owing to its microporous nature, with a Brunauer–Emmett–Teller (BET) surface area of 378 m^2^/g for CIM91. Two different behaviors are observed depending on the ionic liquid used. The BET surface area for [BMIM][Cl]/CIM91 was 348 m^2^/g for an IL loading of 5%; when this load is exceeded, the BET surface area decreases drastically, losing the porosity of the composite. The BET surface areas for [BMIM][PF_6_]/CIM91 decrease with IL incorporation, proportional to the amount of IL loaded.

The relation between the BET surface areas and the ILs loadings was investigated, and a linear relationship was revealed in the [BMIM][PF_6_] materials. The BET surface area decreases linearly as the IL loading increases and the correlation is accurately described with the equation provided in Figure S9. This correlation suggests that the surface area of the [BMIM][PF_6_]/CIM91 samples can be tailored easily by the amount of IL in the structure. This linear behavior is not observed in the [BMIM][Cl]/CIM91 samples, where a drastic surface area decrease is observed. Based on these results, we speculate that most of the [BMIM][X] are inside the CIM91 pores, and as the [BMIM][X] was loaded, the surface area decreased, blocking the pores in the [BMIM][Cl]/CIM91 samples (10 and 20%).

The thermal behavior of the compounds shows that CIM91 has a lower weight percent loss between the beginning of the degradation of the compounds and the final data at 600 °C compared to the [BMIM][Cl]/CIM91 samples and the weight loss slightly increases as the [BMIM][Cl] loading increases. This behavior is not observed so clearly in the case of the [BMIM][PF_6_]/CIM91 samples, with similar thermal behaviors in the [BMIM[PF_6_] (10%) and [BMIM[PF_6_] (20%) synthesis. At the initial stage, the rate of thermal degradation slows down with IL loading in the [BMIM][Cl]/CIM91 samples (Figures S10 and S11)

The CIM91 shows a first weight loss of ca. 19% below 200 °C, attributed to the solvent removal. Another weight loss at 360 °C is associated with the full degradation of the compound. This temperature decreases to around 310 °C and 270 °C when [BMIM][PF_6_] and [BMIM][Cl] are incorporated into the CIM91, respectively. The interaction between the ILs and CIM91 can be responsible for the decrease in the thermal stability of the samples upon IL loadings, as has been previously reported in different imidazolium IL-supported compounds [23,25,26,27].

**Figure 3:**
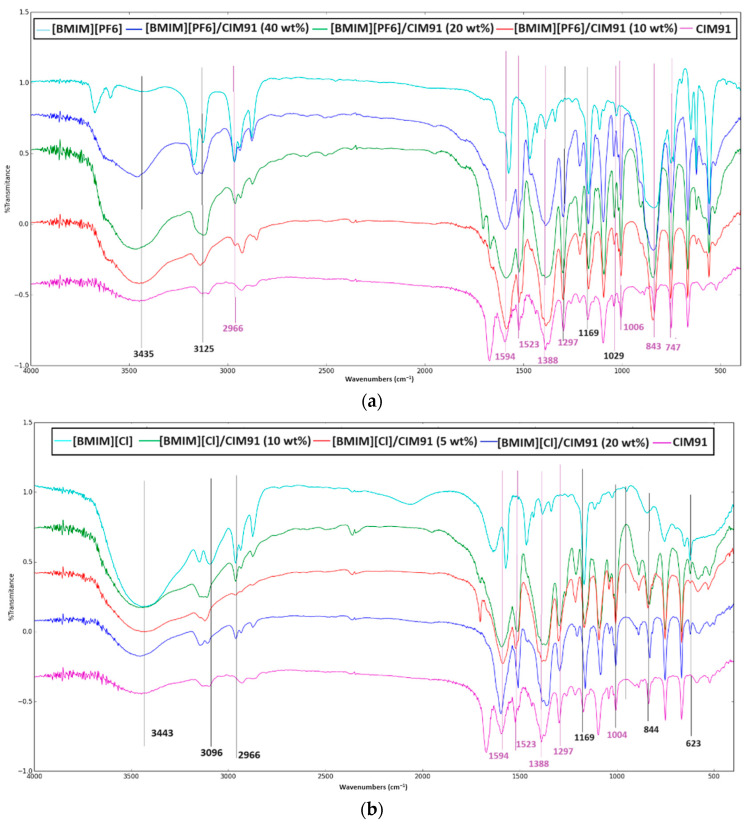
(**a**) IR spectra for CIM91, [BMIM][PF_6_], and [BMIM][PF_6_]/CIM 91 samples for 4000−500 cm^−1^. (**b**) IR spectra for CIM91, [BMIM][Cl], and [BMIM][Cl]/CIM 91 samples for 4000−500 cm^−1^.

The compounds were also characterized by FT-IR spectroscopy. The major IR features of the samples and the corresponding peak assignments were performed according to the literature [28,29,30]. The IR spectra of [BMIM][PF_6_]/CIM91 (5, 10, and 20 wt%) and [BMIM][Cl]/CIM91 (10, 20, and 40 wt%) showed the characteristic peaks of [BMIM][PF_6_] and [BMIM][Cl] as given in Figure 3, and Tables S4 and S5, which proves the successful incorporation of IL into CIM91 [30].

The infrared spectrum of CIM91 contains bands in the region of 1600–1200 cm^−1^, attributed to the carboxylate and trizole ligands. Bands due to the C=N stretching vibrations of triazole are observed at 1523 and 1004 cm^−1^_._ The characteristic bands due to the asymmetric and symmetric vibration modes of the carboxylate groups, with a very strong band at around 1594 cm^−1^, and a moderately strong band at around 1388 cm^−1^ range, respectively, are observed in all the IR spectra.

The presence of the aliphatic asymmetric and symmetric C-H stretching vibrations bands at 2966 and 2875 cm^−1^ and the presence of the characteristic IR stretching bands at 1169, 842, and 624 cm^−1^ confirms the presence of imidazolium cations in the IR spectra. The peaks at 840 and 740 cm^−1^ result from the asymmetric and symmetric stretching modes of the PF_6_^−^ anions of the [BMIM][PF_6_] compound [23].

Upon the formation of the [BMIM][PF_6_]/CIM91 and [BMIM][Cl]/CIM91 compounds, the characteristic peaks of both the CIM91 and [BMIM][X] compounds are presented, indicating that both materials were intact after the incorporation. Moreover, as the [BMIM][X] loadings increase, the peaks assigned to [BMIM][X] are intensified. However, the data revealed some changes in either the position or the intensity of some bands, suggesting the presence of direct interactions between CM91 and [BMIM][X] (Figures S12 and S13, Tables S4 and S5)

#### 2.1.1. Structural Studies

**Figure 4:**
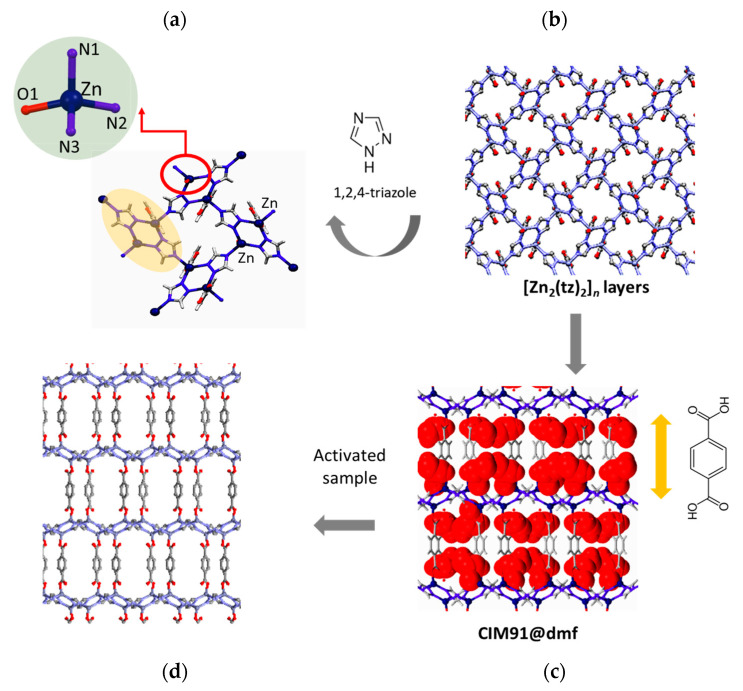
(**a**) Coordination environments of Zn^II^ metal atoms. (**b**) Structure of [Zn_2_(tz)_2_]*_n_* layers (**c**,**d**) Three-dimensional pillared-layered framework with (**c**) and without (**d**) guest solvent molecules.

CIM91 was isolated as a single crystal and its structure was previously reported by Zizhu Yao et al., [24]. The combination of the N-donor triazole ligand with Zn(II) ions composed the two-dimensional grids, which are interconnected in the third dimension by the pillar dicarboxylate linker. Both ligands act as anionic units. A schematic view of the compound is shown in Figure 4.

This compound is a pseudo-polymorph of the previous CIM81 [7,8]. The different supramolecular isomers of the general formula ^∞^_3_Zn_2_tz_2_(bdc).X (X = DMA or DMF) crystallize in the tetragonal P*4/ncc* (CIM81) and orthorhombic P*nma* (CIM91) space groups. The solvents act as structural agents that lead to the formation of the different polymorphs.

Each Zn (II) ion is in a distorted tetrahedral geometry surrounded by three nitrogen atoms and one carboxylate oxygen atom from one bdc^−2^ linker. The triazolate ligands bind to three Zn ions through a typical μ1,2,4-bridging fashion, while the bdc^−2^ ligands coordinate with two Zn ions. The adjacent Zn (II) ions in the 1,2-positions from two tz- anions create binuclear Zn_2_ units with the layers. These 2D layer motifs are further connected by linear dicarboxylate pillars to generate the final 3D pillared-layered structure. The connection of 2D nets by pillars results in 3D frameworks with a pcu topology.

#### 2.1.2. CO_2_ Adsorption Studies

The CO_2_ adsorption properties of CIM91 and the composite materials were evaluated in volumetric equipment at 298 K and 318 K between 0 and 5.5 bar of pressure. The CO_2_ uptake increases with pressure and all the compounds displayed a reversible type I microporous adsorption isotherm (Figure S14–S16). As is evident, there is no hysteresis loop, discarding any strong bonding between the CO_2_ and the sole MOF, nor diffusion restrictions of CO_2_ molecules in its porous system. CIM91, [BMIM][Cl]/CIM 91 (20, 10, and 5 wt%), and [BMIM][PF_6_]/CIM 91 (40, 20, and 10 wt%) showed CO_2_ uptakes at 5.5 bar with saturations between 21.4 cm^3^g^−1^ and 50.8 cm^3^g^−1^ at 298 K, and 16 cm^3^g^−1^ and 39.2 cm^3^g^−1^ at 318 K in [BMIM][Cl]/CIM91 (20 wt%) and [BMIM][PF_6_]/CIM 91 (20 wt%), respectively. (Table 1). It is noteworthy that CO_2_ isotherms for CIM91 were performed twice at both temperatures (298 and 318 K), obtaining in both cases similar results. So, we could confirm not only the repeatability of the adsorption analysis but also the material stability.

In general, the CO_2_ uptake capacity remained similar or slightly decreases compared to the pristine CIM91 as the IL loading was increased, except for the [BMIM][PF_6_]/CIM91 (20 wt%) sample where it increased at both 298 K and 318 K compared to CIM91. Moreover, the [BMIM][PF_6_]/CIM91 composites have better CO_2_ adsorption capacity values than the [BMIM][Cl]/CIM91 composites, which can be related to the presence of fluorinated anions that create new and stronger adsorption sites for CO_2_ molecules. It is noteworthy that the surface area of CIM91 was reduced with the addition of [BMIM][X], meaning that the number of adsorption sites is also diminished, causing a decrease in the CO_2_ uptake capacity as well [31,32,33]. However, our results suggest new adsorption sites are created by incorporating the [BMIM][PF_6_] species into CIM91 [26].

Aki et al. measured CO_2_ solubility in imidazolium-based ILs with different anions and various alkyl chains on the cations. They found that CO_2_ solubility is marginally influenced by the alkyl chain length but is strongly affected by the anion [33,34]. This can explain the higher uptake capacity observed for [BMIM][PF_6_]/CIM91 compared to the [BMIM][Cl]/CIM91 samples.

Table 1 summarizes the CO_2_ adsorption capacity of the studied materials and other well-known MOFs with their corresponding MOF-IL at different temperatures. Unlike what was observed in this study, all the composites maintain a certain degree of surface area when the MOF-IL compounds are formed. However, their absolute CO_2_ adsorption capacity is similar to that presented in this study; for example, UiO66, with a CO_2_ uptake of 45 at 303 K, and UiO67 and UiO67-LI with values around 22.9 at 298 and 48.6 at 273 K, respectively.

**Table 1:** CO_2_ adsorption capacity of the studied materials and other well-known MOFs from the literature at 298 K and 318 K.

Compound	BET (m^2^ g^−1^)	CO_2_ Uptake (cm^3^ g^−1^)	T (K)	Qst (kJ/mol)	Ref
CIM91	378	48.18	298	23	This work
		37.24	318		
[BMIM][Cl] (20 wt%)	non-porous	21.43	298	34	This work
		16.99	318		
[BMIM][Cl] (10 wt%)	non-porous	40.95	298	36	This work
		33.26	318		
[BMIM][Cl] (5 wt%)	348	33.32	298	27	This work
		33.60	318		
[BMIM][PF_6_] (40 wt%)	15	31.32	298	27	This work
		24.17	318		
[BMIM][PF_6_] (20 wt%)	27	50.76	298	55	This work
		39.23	318		
[BMIM][PF_6_] (10 wt%)	86	38.26	298	39	This work
		34.03	318		
Zn_2_(TRZ)_2_(BPDC)	470	17.02	298	29.5	[34]
UiO67	2113	22.9	298	16	[35]
		48.6	273		
UiO67-IL	846	22.4	298	26	[35]
		42,7	273		
[BMIM][BF_4_]/MIL-53(Al)	28.26	70.6	293	31	[36]
MIL53(Al)	472.7	161.3	293	27	[36]
ZIF-8	1768	10.32	298	-	[37]
[BMIM][Tf_2_N]_8_@ZIF-8	1639	15.30	298	-	[37]
[C_4_MIM]_2_[NiCl_4_]@ZIF-8	768	40	300	-	[38]
UiO66	838	79	273	-	[39]
		45	303		

The heat of adsorption (Qst) for these composites was determined using the CO_2_ adsorption isotherms at 25 and 45 °C using the Clausius–Clapeyron equation (Figure S17). The Qst value for the sole CIM91 ([Zn_2_(tz)_2_(bdc)]) was stable approximately at −23 kJ/mol, which corresponded to the lowest value determined for all the materials. A significant increase in the heat of adsorption was observed when the ionic liquids were included in the materials. In the case of the [BMIM][Cl] composites, the highest Qst value was found for [BMIM][Cl] (10 wt%) with 36 kJ/mol followed by [BMIM][Cl]( 5 wt%) and [BMIM][Cl] (20 wt%) with values of 34 and 27 kJ/mol, respectively. Meanwhile, the Qst values estimated for the [BMIM][PF_6_] composites were in general superior to those for the other composites, showing values of 55, 39, and 27 kJ/mol for [BMIM][PF_6_] (20 wt%), [BMIM][PF_6_] (10 wt%), and [BMIM][PF_6_] (40 wt%), respectively. These greater values indicate stronger interactions of the CO_2_ molecules with the solid materials compared to the sole MOF due to the presence of the fluorinated anion in the ionic liquid, as previously reported in the literature [40]. In this case, despite showing higher isosteric heat of CO_2_ adsorption for the samples containing the IL species compared to the sole CIM91 MOF, it does not have an evident effect over the regenerative capacity, since there is no hysteresis loop, with no restrictions in the desorption process. It is noteworthy for both cases that the high loadings of the ionic liquid on the MOF support hinder the host–guest interaction (CO_2_–composite) owing to the steric hindrance generated inside the cavities of the solid materials [41]. In addition, the isosteric heat of CO_2_ adsorption values are between 23 and 55 kJ/mol, so it can be considered as a physisorption phenomenon, still lower than that of most chemically active soluble adsorbents such as aqueous amines (>70 kJ/mol) [42] and amino-functionalized materials (up to 98 kJ/mol) [43].

#### 2.1.3. Dye Adsorption

Different IL/MOF composites have been used to remove methylene blue (MB) dye from different solutions [44,45,46,47]. However, much work is still required to reach molecular-level insights on the structure–performance relationships needed for the rational design of new composite materials [48]. In this sense, the adsorption capabilities of the composites for removing MB in water and ethanol have been studied (Table S6, Figures S17–S38). The time-dependent adsorption quantity of MB on CIM91 and composites are represented in Figures S39–S42. The dye was prepared in different concentrations (1, 5, 10, and 25 ppm in 20 mL of the corresponding solvent). The variation in the absorbance of the different concentrations with time was obtained. For both solvents, the signal obtained with a concentration of 10 ppm is the optimal one for carrying out the study since it prevents the measurement from saturating, distorting the pattern, and making the signal too weak to be measured. Next, the variation in absorbance as a function of time was obtained by introducing the composites into the dye solution. To prepare the samples, 20 mg of compound in 20 mL of solution were used. The changes in concentration for these samples were evaluated by UV–visible spectra. The entire absorption spectrum is shown and a representation of the intensity of the normalized absorbance maximum as a function of time is also introduced (Figures S17–S36).

The quantity and efficiency of MB could be calculated by the equations Q = (C_0_ − C_t_)V/m and efficiency (%) = (C_0_ − C_t_) × 100/C_0_, respectively, where C_0_ and C_t_ represent the concentration of the dye at the beginning and at a certain time (mg L^−1^) and m and V are the adsorbent quality (g) and the dye volume (L), respectively. (Table S6)

Considering the poor water stability of [BMIM][Cl]/CIM91, the dye-adsorption properties in ethanol solution were also investigated. Data showed that the [BMIM][Cl]/CIM91 (x%) compounds (x = 5, 10, and 20%) show similar behavior to the CIM91 in water, absorbing 50% of the MB in 24 h (Figures S24–S27).

**Figure 5:**
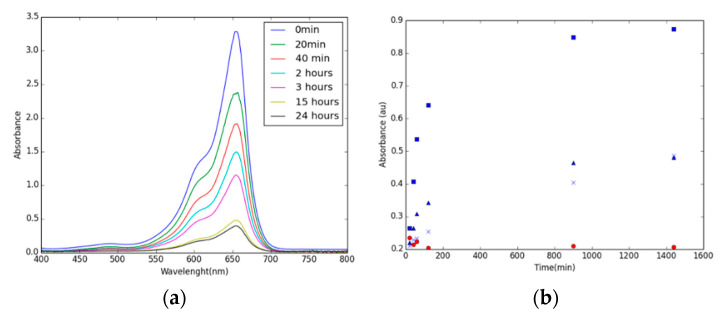
(**a**) UV-visible spectra of MB (10 ppm) absorbed by [BMIM][Cl]/CIM91(10%) in ethanol. (**b**) Maximum absorbance of pristine CIM91 (circle), [BMIM][Cl]/CIM91 (5%) (triangle), [BMIM][Cl]/CIM91 (10%) (square), and [BMIM][Cl]/CIM91 (20%) (cross) in ethanol over time.

However, the performance of the [BMIM][Cl]/CIM91 in the ethanol solvent was much more promising. This composite absorbs the same amount of MB (6%) as the pristine CIM91, in less than 20 min, and after 15 h, 80% of the MB was absorbed (Figure S33–S36). Furthermore, the MB solution in the [BMMIM][Cl]/CIM 91 (10%) sample becomes almost colorless after 24 h with a removal efficiency of 86,5% (Figure 5). [BMIM][Cl]/CIM91 (5%) shows a lower removal efficiency than the other compounds, needing more than 3 h to absorb 16% of the MB and reaching a maximum of 33% in 24 h.

**Figure 6:**
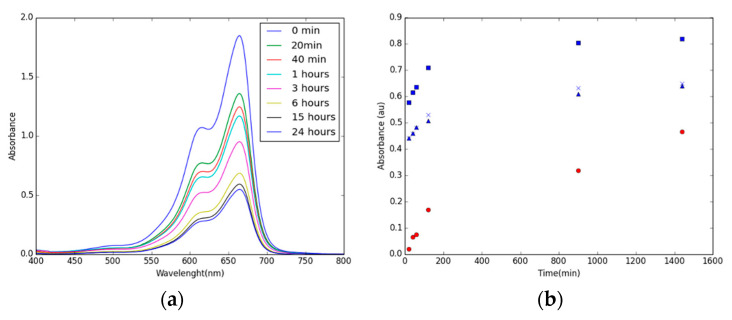
(**a**) UV-visible spectra of MB (10 ppm) absorbed by [BMIM][PF_6_]/CIM91(20%) in water. (**b**) Maximum absorbance of pristine CIM91 (circle), [BMIM][PF6]/CIM91 (10%) (triangle), [BMIM][PF6]/CIM91 (20%) (square), and [BMIM][PF6]/CIM91 (40%) (cross) in water over time.

[BMIM][PF_6_]/CIM91 (20%) in water solvent required only three hours to reach a level at which the pristine CIM91 saturates 50%. After 24 h of immersion, it is capable of absorbing around 70% of the dye load present in the solution. On the other hand, [BMIM][PF_6_]/CIM91 (10% and 40%) are not able to reach the absorption values of the pristine CIM91, reaching 36% and 40%, respectively (Figure 6). [BMIM][PF_6_]/CIM91 (x%) and CIM91 compounds barely absorbed MB when ethanol was used as a solvent.

The data illustrated a significant improvement in the adsorption rate of the parent CIM91 upon IL incorporation, [BMIM][Cl] in the ethanol solvent, and [BMIM][PF_6_] in water.

### 2.2. Remarks

Molecular modeling could be a powerful tool to elucidate the adsorption sites of the different molecules onto the different materials. Previous studies have shown that interactions between MOFs and ILs lead to new adsorption sites with different gas affinities compared to their counterparts in pristine MOFs [49]. Moreover, the incorporation of ILs into MOFs will change the pore environment, and usually, the pore size has a direct effect on the separation performance of adsorbents [50].

Due to the confinement effect, both the cations and anions of ILs in IRMOF-1 are more packed compared with the bulk phase. In this example, the anion has a stronger interaction than the cation with IRMOF-1. The small [BF_4_]^−^, [PF_6_]^−^, and [SCN]^−^, particularly the former two, were located preferentially near the metal cluster of IRMOF-1. However, the bulky and chain-like [Tf_2_N]^−^ and [BMIM]^+^ reside proximally to the phenyl ring [33]. Ab initio molecular dynamics simulations and DFT calculations were performed in IRMOFs and they concluded that spherical anions, e.g., Br^−^ and PF_6_^−^ can stabilize the frameworks more effectively than elongated anions [51]. Atomistic simulations were also used to identify the interfacial interaction between different ILs and ZIF-8 and their effect on selective CO_2_ capture. This study proved that the nature of the anions is more important than cations to enhance CO_2_ selectivity. Anions such as [BF_4_]^−^, [Tf_2_N]^−^, and [PF_6_]^−^ improve the selectivity and CO_2_ separation, which is also confirmed by the enhanced heat of adsorption for CO_2_ [52].

## 3. Materials and Methods

### 3.1. Synthetic and Activation Procedures

Starting materials, 1,2,4-triazol (98%) (Htz), benzene-1,4-dicarboxylic acid (98%) (H_2_bdc), zinc nitrate hexahydrate (98%), 1-Butyl-3-methylimidazolium hexafluorophosphate ([BMIM][PF_6_]), 1-Butyl-3 methylimidazolium chloride ([BMIM][Cl]), and solvents were supplied from Sigma-Aldrich (Steinheim, Germany).

Acetone (99.8%, Scharlau, Barcelona, Spain), methanol (99.9%, Sigma Aldrich, Barcelona, Spain), and tetrahydrofuran (99.9%, Sigma Aldrich, Barcelona, Spain) were used as the solvents for the IL dissolution and to promote their impregnation into the MOF structure. For the dye absorption studies, methylene blue was supplied by Panreac (Barcelona, Spain). Ultrapure deionized water was obtained using the Milli-Q gradient A10 water purification system from Millipore (Watford, UK). The 45 mL Teflon solvothermal reactor and the stainless-steel autoclaves for the synthesis of MOF were supplied by Parr Instrument Company (Moline, IL, USA).

### 3.2. Synthetic and Activation Procedures

[Zn_2_(tz)_2_(bdc)]·solv (CIM91)

A mixture of Zn(NO_3_)_2_·6H_2_O (592 mg, 2 mmol), 1,2,4-triazole (140 mg, 2 mmol), and benzene-1,4-dicarboxylic acid (170 mg, 1 mmol) was dissolved in 15 mL of N,N-dimethylformamide (DMF). The final mixture was placed in a Parr Teflon-lined stainless steel vessel (45 mL) under autogenous pressure and heated at 120 °C for 72 h. The colorless crystals obtained were filtered by gravity and dried at 80 °C. The guest molecules were removed by a two-step activation procedure. The sample was immersed in acetone for 24 h to remove the non-volatile solvates (this process was repeated two times) followed by a pore evacuation at 100 °C. The empty-pore forms of the compound were used for the following synthetic procedures.

Yield: 83%. Anal. calc. for C_12_H_8_N_6_O_4_Zn_2_ (in%): C 33.44, H 1.87, N 19.50; Found: C 33.06, H 1.90, N 19.26. IR (cm^−1^): 3450m, ν(OH) + ν(NH); 1594s, 1523m, 1388m, ν_asym_(CO_2_) + ν_sym_(CO_2_) + ν(CC,CN).

[BMIM][PF_6_]/CIM91 (20 wt%)

A total of 29 µL of [BMIM][PF_6_] was dissolved in 15 mL of acetone in a Schlenk flask. Activated CIM91 (0.16 g) was added to the solution, and the resulting mixture was continuously stirred at r.t. for 3 h. The solvent was evaporated, and the resulting sample was dried in a vacuum oven at 70 °C overnight. The resultant white solid was labeled as [BMIM][PF_6_]/CIM91 (20 wt%) according to its weight percent.

The other samples with 10 and 40 wt% loadings of [BMIM][PF_6_] were prepared similarly with 7 µL and 80 µL, respectively.

[BMIM][Cl]/CIM91 (20 wt%)

In total, 40 mg of [BMIM][Cl] was dissolved in 15 mL of acetone in a Schlenk flask. Activated CIM91 (0.16 g) was added to the solution, and the resulting mixture was continuously stirred at r.t. for 3 h. The solvent was evaporated, and the resulting sample was dried in a vacuum oven at 70 °C overnight. The resultant white solid was labeled as [BMIM][Cl]/CIM91 (20 wt%) according to its weight percent.

The other samples with 10 and 5 wt% loadings of [BMIM][Cl] were prepared in a similar manner with 20 mg and 8 mg, respectively.

### 3.3. Instrumentation

The X’Pert Diffractometer supplied by PANalytical and operating with Bragg–Brentano geometry was used. The data collection was carried out using Cu K1 radiation (λ = 1.5418 Å) over the angular range from 5.01° to 80.00° (0.02° steps) with a total exposure time of 30 min.

The IR spectra (450–4000 cm^−1^) were recorded on a powder sample by means of a Shimadzu IRAffinity1 spectrophotometer equipped with a Pike technologies GladiATR.

The nitrogen adsorption isotherms were measured on a Gemini V 2365 Model supplied by Micromeritics (Norcross, GA, USA) surface area analyzer at 77 K in the range 0.02 ≤ P/P_0_ ≤ 1.00. For each measurement, approximately 100 mg of the sample was used, and the samples were activated under vacuum in two steps at 90 and 100 °C for 1 and 24 h, respectively. After the activation, the sample was cooled to 77 K using liquid nitrogen, and a free space measurement was performed with helium gas. The Brunauer, Emmett, and Teller (BET) method was used to calculate the surface area.

The thermogravimetric analysis was carried out in a Perkin-Elmer Pyris Diamond TG/DTA thermal analyzer (Perkin-Elmer, Waltham, MA, USA), typically using a few mg of the samples placed on an alumina crucible under a nitrogen atmosphere at a flow rate of 20 cm^3^·min^−1^. The temperature was ramped from 25 to 250 °C and at a heating rate of 5 °C min^−1^.

The elemental analyses (C, H, N) were carried out with a CNHS FLASH EA 1112 elemental analyzer (Thermo Fisher Scientific, Waltham, MA, USA).

The scanning electron microscopy (SEM) analysis of the microcrystalline materials was performed using an EVO 15 microscope (ZEISS, Jena, Germany).

A UV-2450 spectrophotometer (SHIMADZU, Tokyo, Japan) was used to measure, as a function of wavelength, the amount of light transmitted after passing through the sample solution. The samples were activated and dried under a high vacuum before the dye adsorption studies.

The adsorption–desorption isotherms of pure CO_2_ were collected in a volumetric analyzer type VTI HPVA-100 Scientific Instrument (VTI Scientific, Stockholm, Sweden). Approximately 100 mg of the materials were previously evacuated in situ at 110 °C under vacuum (9 × 10^3^ bar) for 12 h. The analysis temperature was fixed using a thermostatic polyethyleneglycol bath. The isotherm equilibrium points were collected considering the following two equilibrium criteria: (i) a pressure drop below 0.2 mbar in 3 min or (ii) a maximum equilibrium time of 60 min. The CO_2_ adsorption equilibrium points at 298 y 318 K were fitted to the Sips equation (Sips, 1948).

## 4. Conclusions

A zinc pillared-layered MOF was solvothermally synthesized and various masses of [BMIM][X] were supported on CIM91 MOF. The resulting composites were characterized by X-ray powder diffraction (XRD), FT-IR spectra, scanning electronic microscopy (SEM), Energy dispersive X-ray (EDX) analysis, N_2_ adsorption measurements, and thermal analysis. The adsorption of the methylene blue organic dye and the CO_2_ uptake were investigated. The results revealed that the incorporation of [BMIM][X] into the structure of CIM91 at different loadings influences the adsorption properties of the MOF.

A series of CO_2_ adsorption–desorption analyses were conducted on all materials at 298 K and 318 K. The results demonstrated a significant enhancement in the CO_2_ adsorption properties of the sole MOF, CIM91, particularly when the [BMIM][PF_6_] species were included in its structure, which exhibited a double isosteric heat of CO_2_ adsorption. They also demonstrated higher CO_2_ uptake than other well-known MOFs used with success, such as ZIF-8, or UiO-67.

The data showed that [BMIM][X] incorporation considerably reduces the time required for reaching 50% MB removal concerning CIM91, from 24 h to 3 h (in [BMMIM][Cl]/CIM 91 (10%) and [BMIM][PF_6_]/CIM91 (20%)). The results highlight that IL incorporation offers a more efficient prospect than the pristine CIM91 in MB removal, resulting in 70% for [BMIM][PF_6_]/CIM91 (20%) in water and 86% for [BMMIM][Cl]/CIM 91 (10%) in ethanol. The amount of [BMIM][X] incorporated into the CIM91 seems to be crucial for the optimization of adsorption properties. The change in anion in [BMIM][X] has offered an opportunity to tailor the physicochemical properties of the final composites. The presence of fluoroanions has been particularly suitable for CO_2_ storage and MB removal. This work provides new insights for the future design and assembly of IL/MOF composites.

## Data Availability

The original contributions presented in the study are included in the article/Supplementary Material, further inquiries can be directed to the corresponding author.

## Supplementary Materials

The following supporting information can be downloaded at: https://www.mdpi.com/article/10.3390/molecules29153644/s1. Figure S1. (A). X-Ray diffraction patterns of CIM-91 before and after the activation process. (B). Comparison (FULLPROF) of powder X-ray diffractogram obtained for Cim91 and the calculated from single crystal data. Figure S2. (A). X-ray diffraction patterns of [BMIM][Cl]/CIM91 (40 wt%), [BMIM][Cl]/CIM91 (20 wt%), [BMIM][Cl]/CIM91 (10 wt%), and [BMIM][Cl]/CIM91 (5 wt%) compounds. (B). Comparison (FULLPROF) of powder X-ray diffractogram obtained for (a) [BMIM][Cl]/CIM91 (5 wt%), (b) [BMIM][Cl]/CIM91 (10 wt%), and (c) [BMIM][Cl]/CIM91 (20 wt%) and the calculated from single crystal data. Figure S3. (A). X-ray diffraction patterns of CIM91as, CIM91ac, [BMIM][PF_6_]/CIM91 (40 wt%), [BMIM][PF_6_]/CIM91 (20 wt%), and [BMIM][PF_6_]/CIM91 (10 wt%), compounds. (B). Comparison (FULLPROF) of powder X-ray diffractogram obtained for (a) [BMIM][PF_6_]/CIM91 (10 wt%), (b) [[BMIM][PF_6_]/CIM91 (20 wt%), and (c) [BMIM][PF_6_]/CIM91 (40 wt%) and the calculated from single crystal data. Figure S4. SEM image of CIM91 before and after activation in acetone solvent. Figure S5. SEM image of [BMIM][Cl]/CIM91 crystalline materials and Energy dispersive X-ray (EDX) mapping analysis of composites. Figure S6. SEM image of [BMIM][PF_6_]/CIM91 crystalline materials and Energy dispersive X-ray (EDX) mapping analysis of composites. Table S1: EDX analysis of CIM91, [BMIM][PF_6_]/CIM91 and [BMIM][Cl]/CIM91 samples. Table S2: Experimental (Exp) and theoretical (Theo) weight percent of N, C, and H atoms in the different compounds. Figure S7. Nitrogen uptake analysis at 77K of the [BMIM][PF_6_]/CIM91 (40 wt%), [BMIM][PF_6_]/CIM91 (20 wt%), and [BMIM][PF_6_]/CIM91 (10 wt%), materials. Figure S8. Nitrogen uptake analysis at 77K of the [BMIM][Cl]/CIM91 (20 wt%), [BMIM][Cl]/CIM91 (10 wt%), and [BMIM][Cl]/CIM91 (5 wt%), materials. Table S3: BET surface areas calculated from N_2_ adsorption measurements at 77 K. Figure S9. Correlation between BET surface area and [BMIM][PF_6_] loading on CIM91. Figure S10. Thermogravimetric analysis of [BMIM][PF_6_]/CIM91 (40 wt%), [BMIM][PF_6_]/CIM91 (20 wt%), and [BMIM][PF_6_]/CIM91 (10 wt%). Figure S11. Thermogravimetric analysis of [BMIM][Cl]/CIM91 (20 wt%), [BMIM][Cl]/CIM91 (10 wt%), [BMIM][Cl]/CIM91 (5 wt%) and CIM91. Table S4. Characteristics peaks of [BMIM][Cl], CIM 91 and [BMIM][Cl]/CIM 91 (5, 10, 20 wt%). Table S5. Characteristics peaks of CIM 91, [BMIM][PF_6_] and [BMIM][PF_6_]/CIM 91 (10, 20, 40 wt%). Figure S12. FTIR spectroscopy of CIM91, [BMIM][Cl], [BMIM][Cl]/CIM91 (5 wt%), [BMIM][Cl]/CIM91 (10 wt%), and [BMIM][Cl]/CIM91 (20 wt%). Figure S13. FTIR spectroscopy of CIM91, [BMIM][PF_6_], [BMIM][PF_6_]/CIM91 (10 wt%), [BMIM][PF_6_]/CIM91 (20 wt%), and [BMIM][PF_6_]/CIM91 (40 wt%). Figure S14. Single-component adsorption-desorption equilibrium isotherms of CO_2_ in the pristine CIM91 at (a) 298 K and (b) 318 K. Figure S15. Single-component adsorption-desorption equilibrium isotherms of CO_2_ in the [BMIM][PF_6_]/CIM91 samples at 298 K and 318 K. Figure S16. Single-component adsorption-desorption equilibrium isotherms of CO_2_ in the [BMIM][Cl]/CIM91 samples at 298 K and 318 K. Figure S17. Heat of CO_2_ adsorption estimated for all materials using Clausius-Clapeyron equation. Figure S18–S37. UV-visible spectra of the different MB absorptions. Figure S38. Methylene blue absorption by [BMIM][Cl](10%) over time. Figure S39–S42. Time-dependent adsorption quantity of MB in the different studies. Table S6. Quantity value and efficiency of MB for [BMIM][Cl]/CIM91 composite in ethanol and CIM 91 and [BMIM][PF_6_]/CIM91 composite in water.
