# Supplementary material for: [BMIM][X] Ionic Liquids Supported on a Pillared-Layered Metal–Organic Framework: Synthesis, Characterization, and Adsorption Properties
Source: Molecules. 2024 Aug 1;29(15):3644. doi: 10.3390/molecules29153644 (PMC11314118; doi:10.3390/molecules29153644)
Supplement: Supplementary file 1 [file molecules-29-03644-s001.zip › molecules-3107141-supplementary.pdf]

[BMIM][X] Ionic Liquids Supported on a Pillared-Layered Metal–Organic Framework: Synthesis, Characterization, and Adsorption Properties

Supplementary Information

Yaiza Martín-García [1], Jesús Tapiador [2], Gisela Orcajo [2], Juan Ayala [3] and Ana B. Lago [1,*]

[1] Laboratorio de Materiales para Análisis Químico (MAT4LL), Departamento de Química, Unidad Departamental de Química Inorgánica, Universidad de La Laguna (ULL), 38206 San Cristóbal de La Laguna, Spain; alu0100823448@ull.edu.es
[2] Department of Chemical, Energy and Mechanical Technology, Rey Juan Carlos University, Calle Tulipán s/n, 28933 Móstoles, Spain; jesus.tapiador@urjc.es (J.T.); gisela.orcajo@urjc.es (G.O.)
[3] Laboratorio de Materiales para Análisis Químico (MAT4LL), Departamento de Química, Unidad Departamental de Química Analítica, Universidad de La Laguna (ULL), 38206 San Cristóbal de la Laguna, Spain; jayala@ull.edu.es
* Correspondence: alagobla@ull.edu.es; Tel.: +34-922318301

Summary

Tables
Table S1. EDX analysis of materials 7
Table S2. Experimental and theoretical weight percent 7
Table S3. BET Surface areas 9
Table S4. IR peaks of **CIM91** and **[BMIM][Cl]/CIM91** materials 11
Table S5. IR peaks of **CIM91** and **[BMIM][PF$_6$]/CIM91** materials 12
Table S6. Quantity value and efficiency of MB for materials 26

Characterization
Figure S1. PXRD patterns of **CIM-91** 2
Figure S2. PXRD patterns of **[BMIM][Cl]/CIM91** materials 3
Figure S3. PXRD patterns of **[BMIM][PF$_6$]/CIM91** material 4
Figure S4. Scanning Electron Microscopy of **CIM91** 5
Figure S5-S6. EDX mapping analysis of material 6
Figure S7-S8. Nitrogen uptake analysis at 77 K **CIM-81**. Contour plot 8
Figure S9. Correlation between BET surface area and IL loading 9
Figure S10. TGA of **CIM-91** and **[BMIM][Cl]/CIM91** materials 10
Figure S11. TGA of of **CIM91** and **[BMIM][PF$_6$]/CIM91** materials 10
Figure S12-S13. FTIR patterns of materials 13

Adsorption experiments
Figure S14. Adsorption-desorption isotherm of CO_2 in **CIM-91** 15
Figure S15. Adsorption-desorption isotherm of CO_2 in **[BMIM][PF$_6$]/CIM-91** 15
Figure S16. Adsorption-desorption isotherm of CO_2 in **[BMIM][Cl]/CIM-91** 16
Figure S17. Heat of CO_2 adsorption 17
Figure S18-S42. Dye Adsorption of MB 18
Figure S38. Methylene blue absorption by [BMIM][Cl](10%) over time 23

X-ray diffraction patterns

Figure S1A. X-Ray diffraction patterns of CIM-91 before (blue) and after (green) the activation process. The structural data have been deposited in the Cambridge Crystallographic Data Centre (CCDC) with the reference number 1471122 (Database Identifier; BEKQUG) [24]. Space Group: P n m a (62), Cell: a 10.1168(3)Å b 26.4504(10)Å c 9.6256(3)Å, α 90° β 90° γ 90°

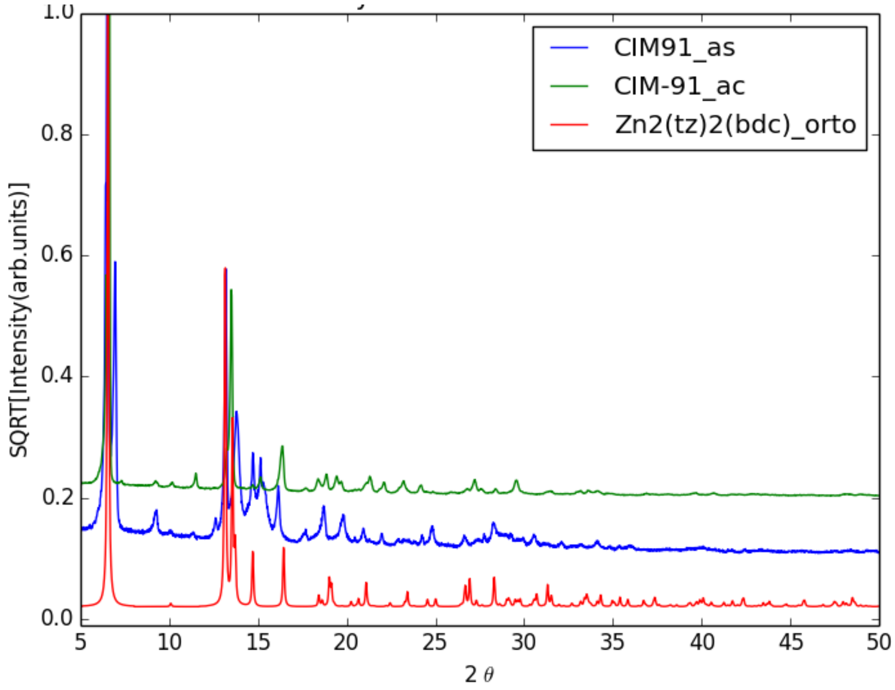

Figure S1B. Comparison (FULLPROF) of powder X-ray diffractogram obtained for **Cim91** and the calculated from single crystal data

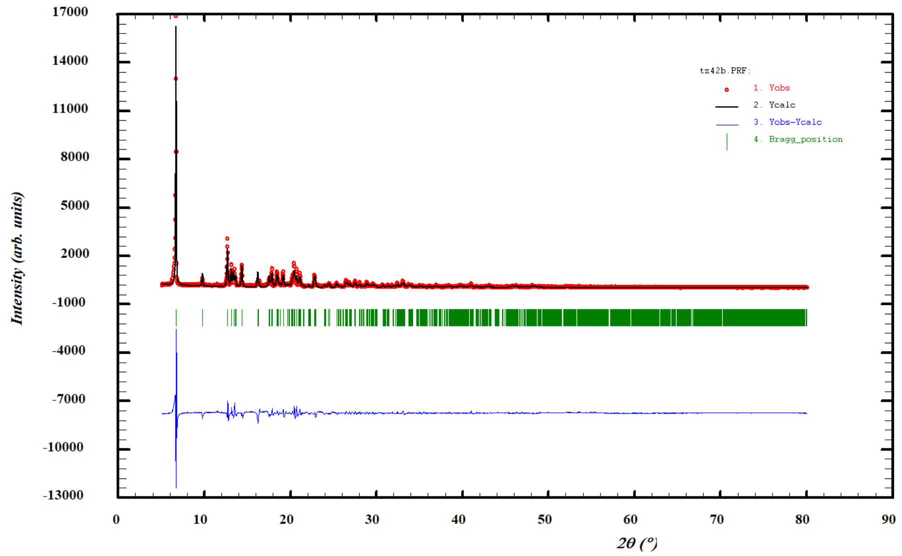

Figure S2A. X-ray diffraction patterns of **[BMIM][Cl]/CIM91 (40 wt%), [BMIM][Cl]/CIM91 (20 wt%), [BMIM][Cl]/CIM91 (10 wt%), and [BMIM][Cl]/CIM91 (5 wt%)** compounds.

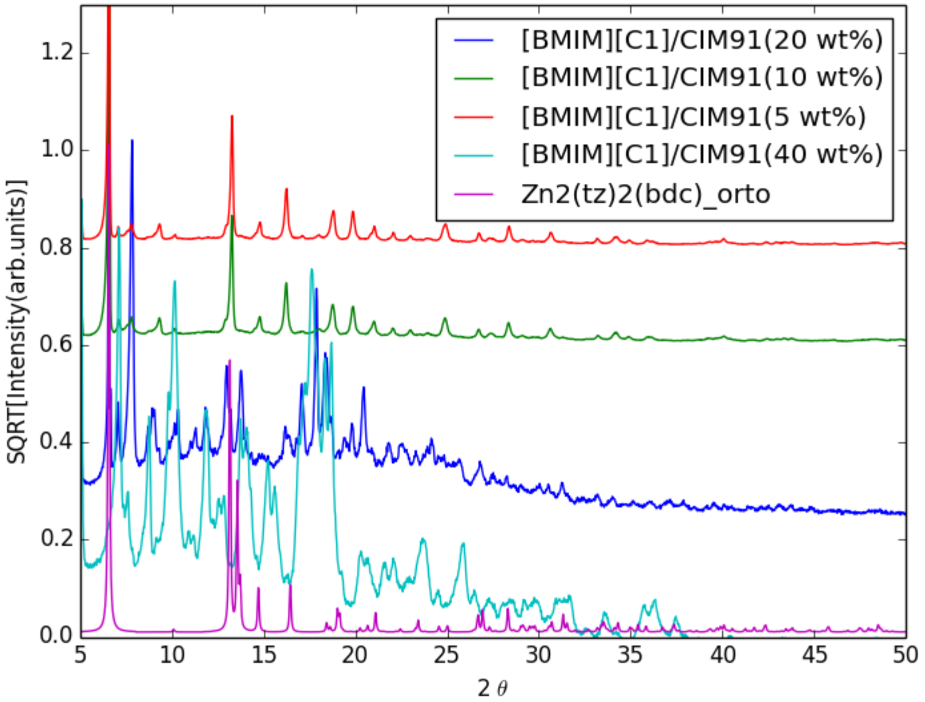

Figure S2B. Comparison (FULLPROF) of powder X-ray diffractogram obtained for a) **[BMIM][Cl]/CIM91 (5 wt%),** b) **[BMIM][Cl]/CIM91 (10 wt%),** and c) **[BMIM][Cl]/CIM91 (20 wt%)** and the calculated from single crystal data.

a)

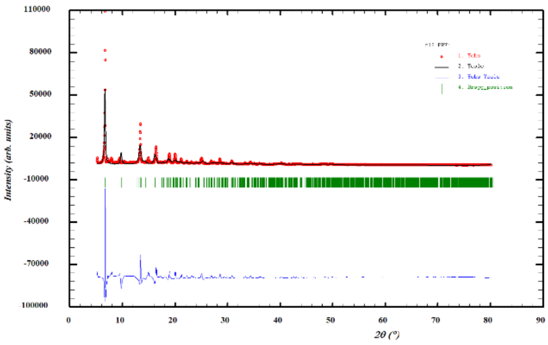

b)

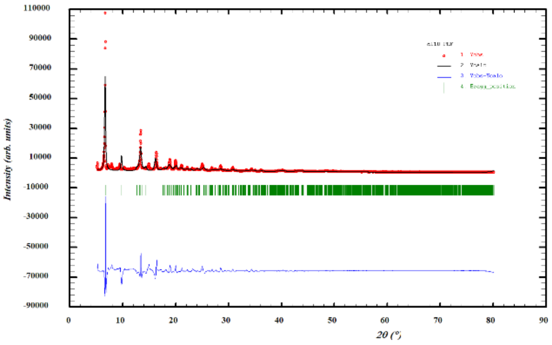

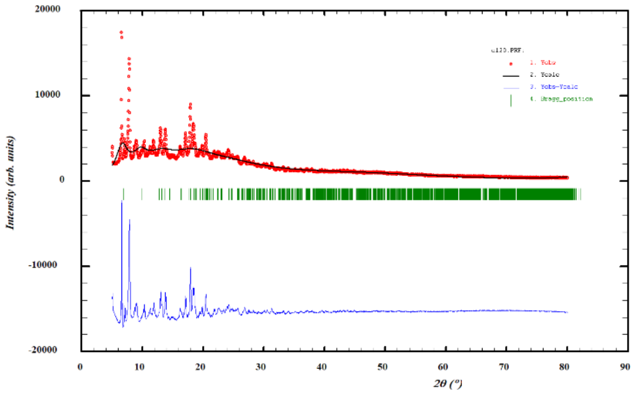

c)

Figure S3A. X-ray diffraction patterns of **CIM91as**, **CIM91ac, [BMIM][PF$_6$]/CIM91 (40 wt%)**, **[BMIM][PF$_6$]/CIM91 (20 wt%),** and **[BMIM][PF$_6$]/CIM91 (10 wt%), compounds**.

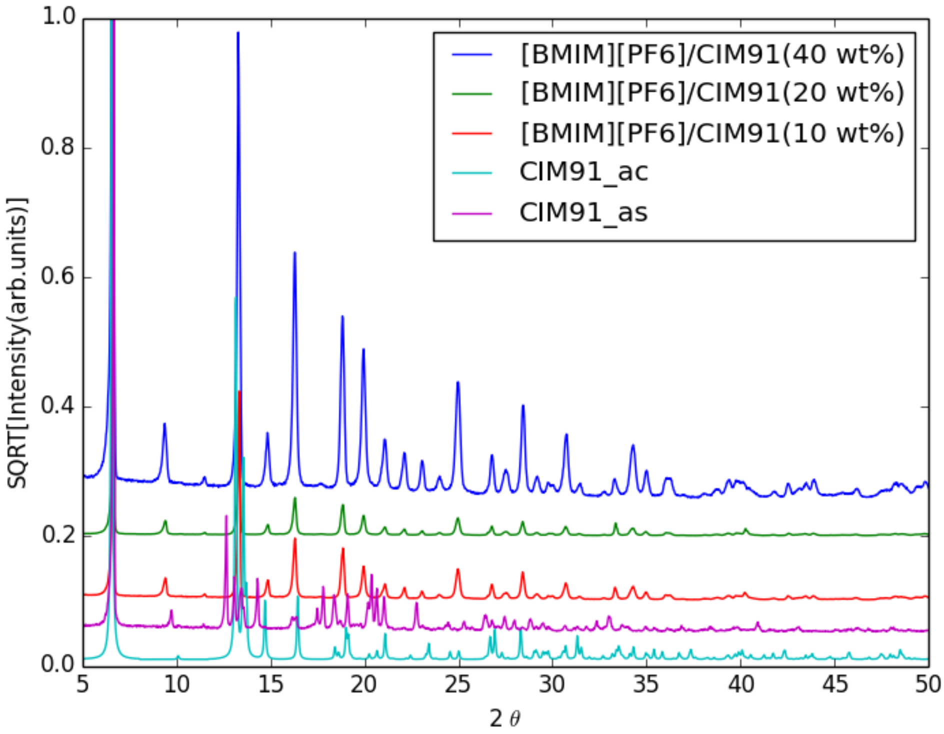

Figure S3B. Comparison (FULLPROF) of powder X-ray diffractogram obtained for a) **[BMIM][PF$_6$]/CIM91 (10 wt%),** b) **[[BMIM][PF$_6$]/CIM91 (20 wt%),** and c) **[BMIM][PF$_6$]/CIM91 (40 wt%)** and the calculated from single crystal data.

a)

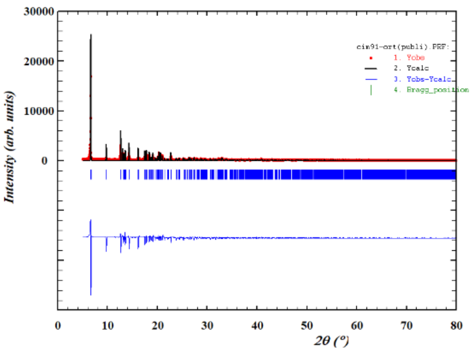

b)

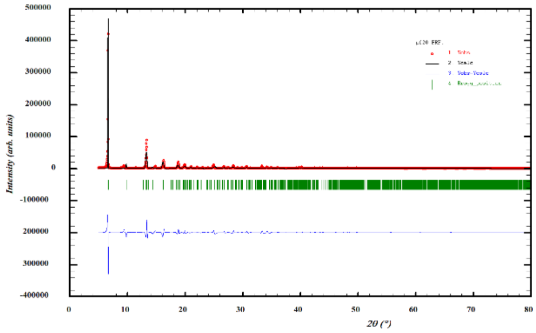

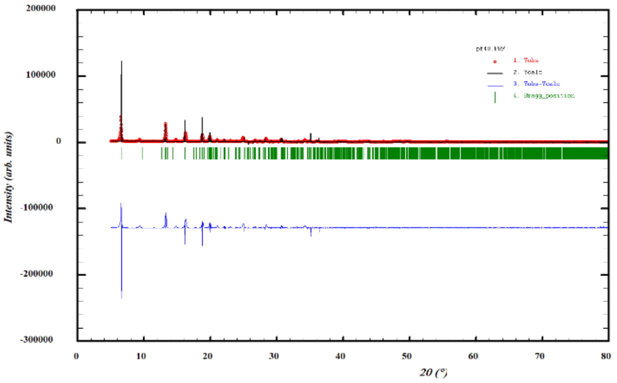

c)

Scanning Electron Microscopy (SEM) images

Figure S4. SEM image of **CIM91** before and after activation in acetone solvent.

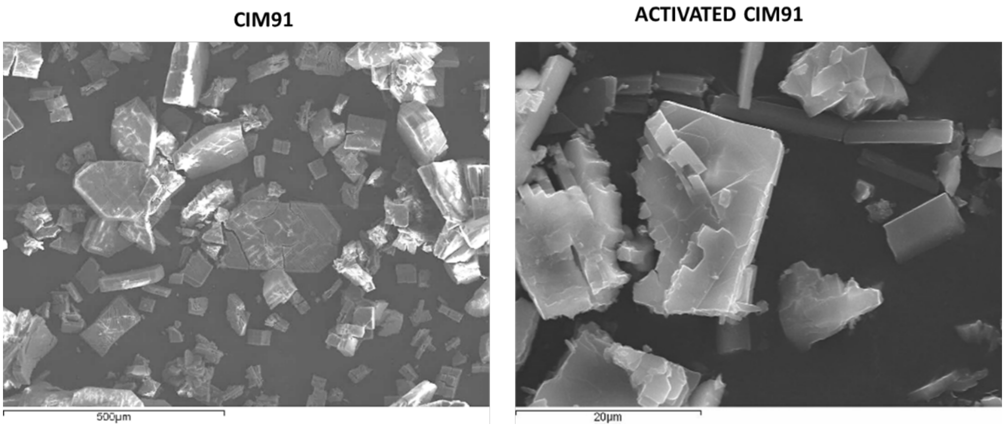

Figure S5. SEM image of **[BMIM][Cl]/CIM91** crystalline materials and Energy dispersive X-ray (EDX) mapping analysis of composites.

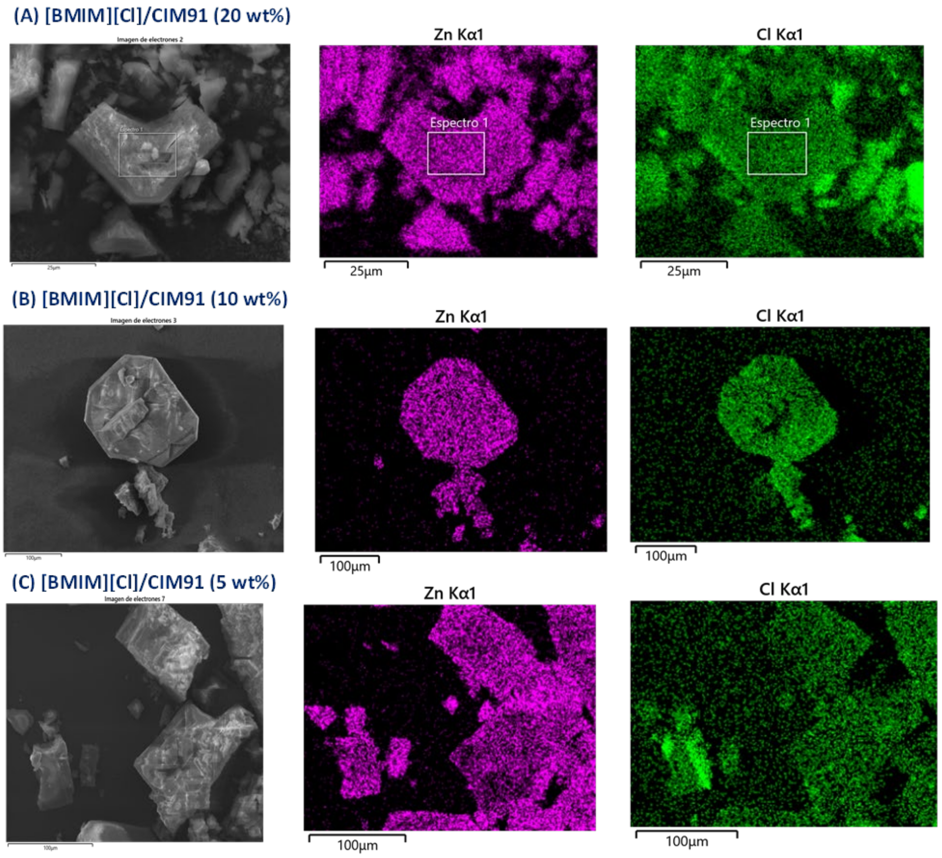

Figure S6. SEM image of **[BMIM][PF$_6$]/CIM91** crystalline materials and Energy dispersive X-ray (EDX) mapping analysis of composites.

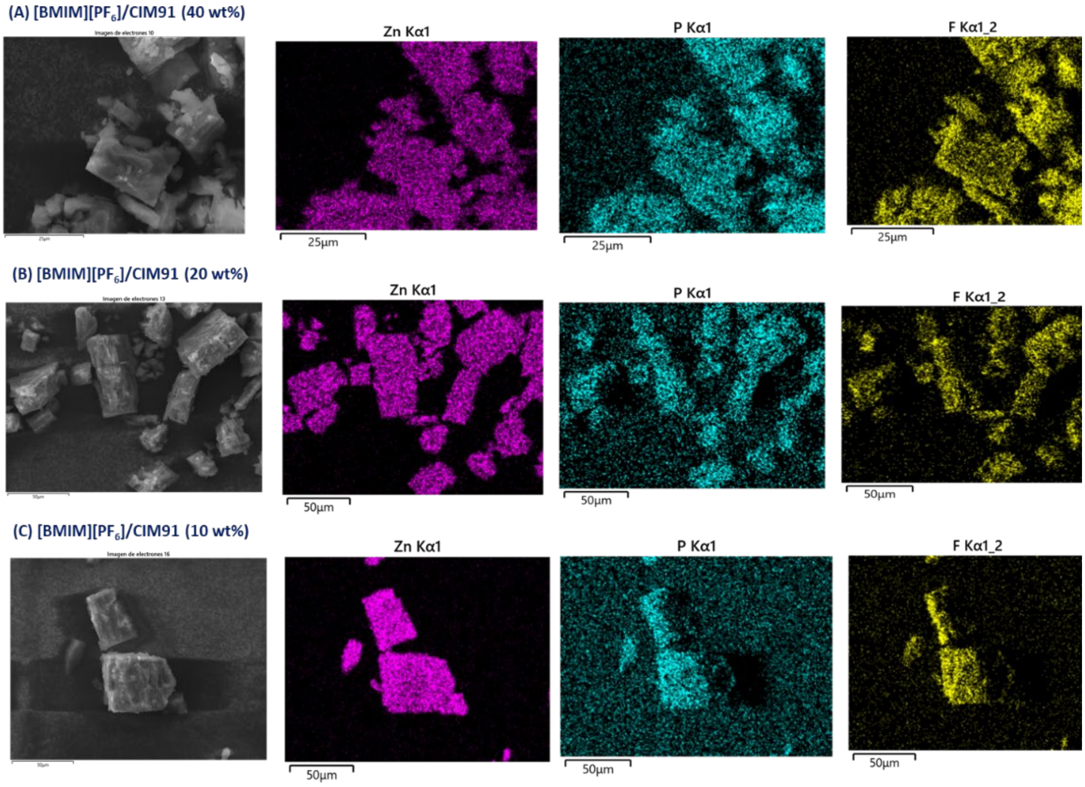

Elemental Analysis

Table S1: EDX analysis of **CIM91**, **[BMIM][PF$_6$]/CIM91** and **[BMIM][Cl]/CIM91** samples.

	**Zn: P**		**Zn:Cl**	
**Compound**	Exp	Theo	Exp	Theo
[BMIM][PF$_6$]/CIM91 (40 wt%)	1:0.39	1:0.65		
[BMIM][PF$_6$]/CIM91 (20 wt%)	1:0.28	1:0.25		
[BMIM][PF$_6$]/CIM91 (10 wt%)	1:0.08	1:0.05		
[BMIM][Cl]/CIM91 (20 wt%)			1:0.53	1:0.4
[BMIM][Cl]/CIM91 (10 wt%)			1:0.25	1:0.2
[BMIM][Cl]/CIM91 (5 wt%)			1:0.07	1:0.05

Table S2: Experimental (Exp) and theoretical (Theo) weight percent of N, C, and H atoms in the different compounds.

	**Carbon (wt%)**		**Nitrogen (wt%)**		**Hidrogen (wt%)**	
**Compound**	Exp	Theo	Exp	Theo	Exp	Theo
$Zn_2(C_2H_2N_3)_2(C_8H_4O_4)$·	33,06	33,44	19,26	19,50	1,90	1,87
[BMIM][PF$_6$]/CIM 91 (40 wt%)	32,20	33,59	14,74	15,67	3,31	3,24
[BMIM][PF$_6$]/CIM 91(20 wt%)	31,55	33,53	14,99	17,10	3,05	2,73
[BMIM][PF$_6$]/CIM 91(10 wt%)	36,40	33,46	14,72	18,90	3,55	2,08
[BMIM][Cl]/CIM 91(20 wt%)	36,51	37,07	16,85	18,90	3,30	3,01
[BMIM][Cl]/CIM 91(10 wt%)	33,46	34,80	16,59	19,28	3,09	2,30
[BMIM][Cl]/CIM 91(5 wt%)	30,35	34,48	16,66	19,32	2,85	2,20

Brunauer–Emmett–Teller (BET) Surface Area Measurements

Figure S7. Nitrogen uptake analysis at 77K of the **[BMIM][PF_6]/CIM91 (40 wt%), [BMIM][PF_6]/CIM91 (20 wt%),** and **[BMIM][PF_6]/CIM91 (10 wt%),** materials.

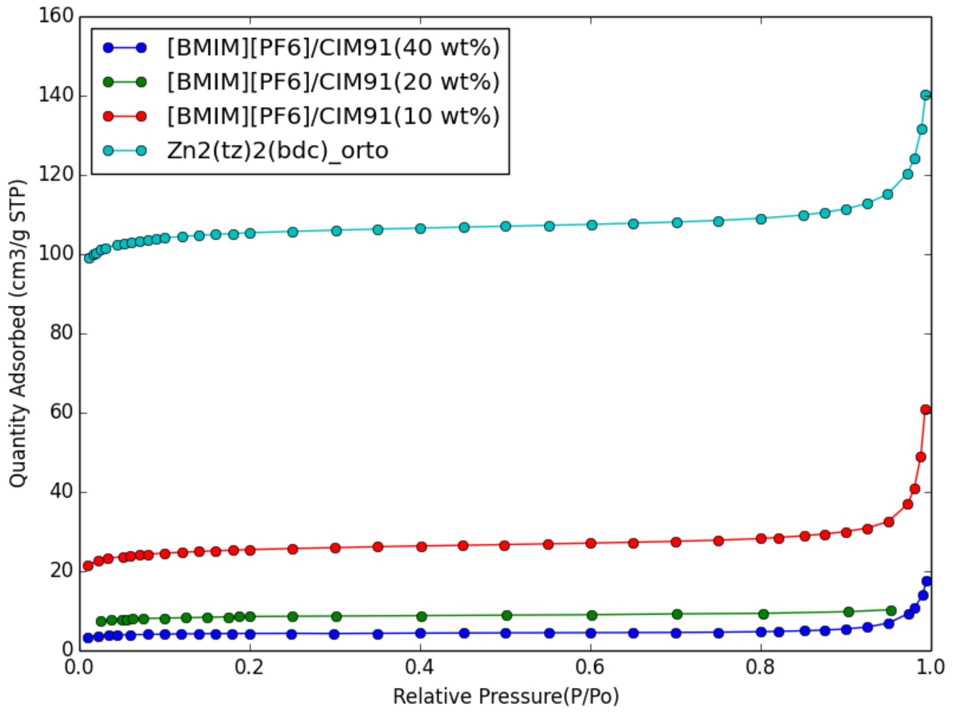

Figure S8. Nitrogen uptake analysis at 77K of the **[BMIM][Cl]/CIM91 (20 wt%), [BMIM][Cl]/CIM91 (10 wt%),** and **[BMIM][Cl]/CIM91 (5 wt%),** materials.

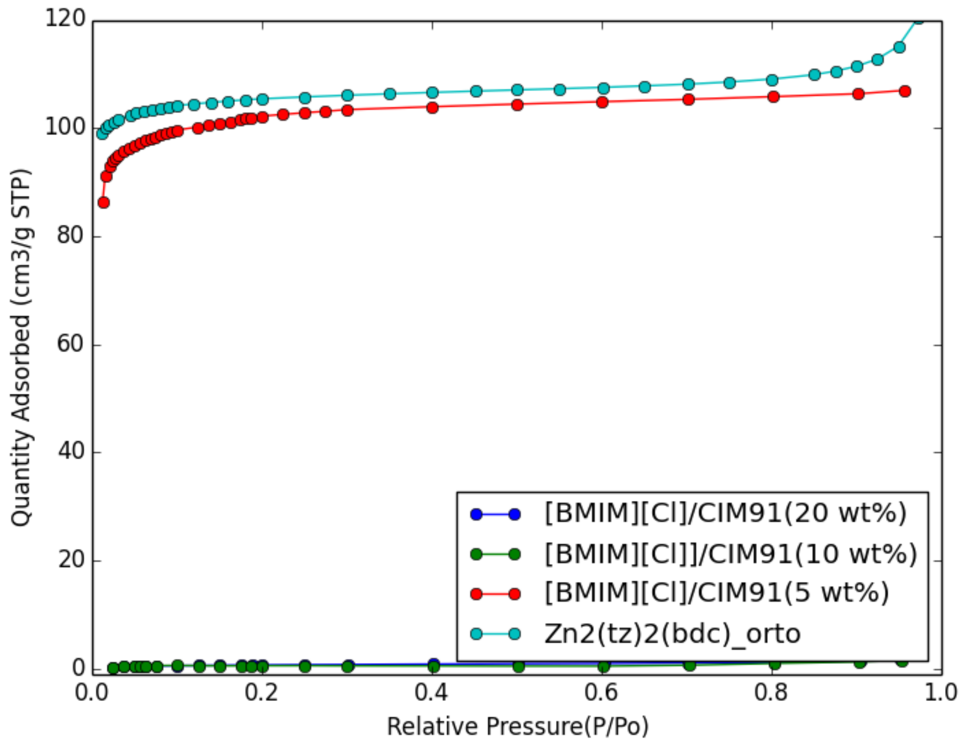

Table S3: BET surface areas calculated from N_2 adsorption measurements at 77 K.

**Compound**	**S_{BET} (m²/g) V_{pore} (cm³/g)**
CIM91	377.8134 m²/g<br>19.7234 Å (pore size)<br>0.186294 cm³/g (pore volume)
[BMIM][PF_6]/CIM91 (40 wt%)	14.6440 m²/g<br>39.5129 Å (pore size)<br>00.014466 cm³/g (pore volume)
[BMIM][PF_6]/CIM91 (20 wt%)	27.2647 m²/g<br>24.7064 Å (pore size)<br>0.015889 cm³/g (pore volume)
[BMIM][PF_6]/CIM91 (10 wt%)	86,4461 m²/g<br>26.4920 Å (pore size)<br>0.057059 cm³/g (pore volume)
[BMIM][Cl]/CIM91 (20 wt%),	2.38 m²/g<br>41.9720 Å (pore size)<br>0.002491 cm³/g (pore volume)
[BMIM][Cl]/CIM91 (10 wt%),	1.5950 m²/g<br>57.7069 Å (pore size)<br>0.002301 cm³/g (pore volume)
[BMIM][Cl]/CIM91 (5 wt%),	348.1240 m²/g<br>15.320 Å (pore size)<br>0.133331 cm³/g (pore volume)

Figure S9. Correlation between BET surface area and [BMIM][PF_6] loading on CIM91

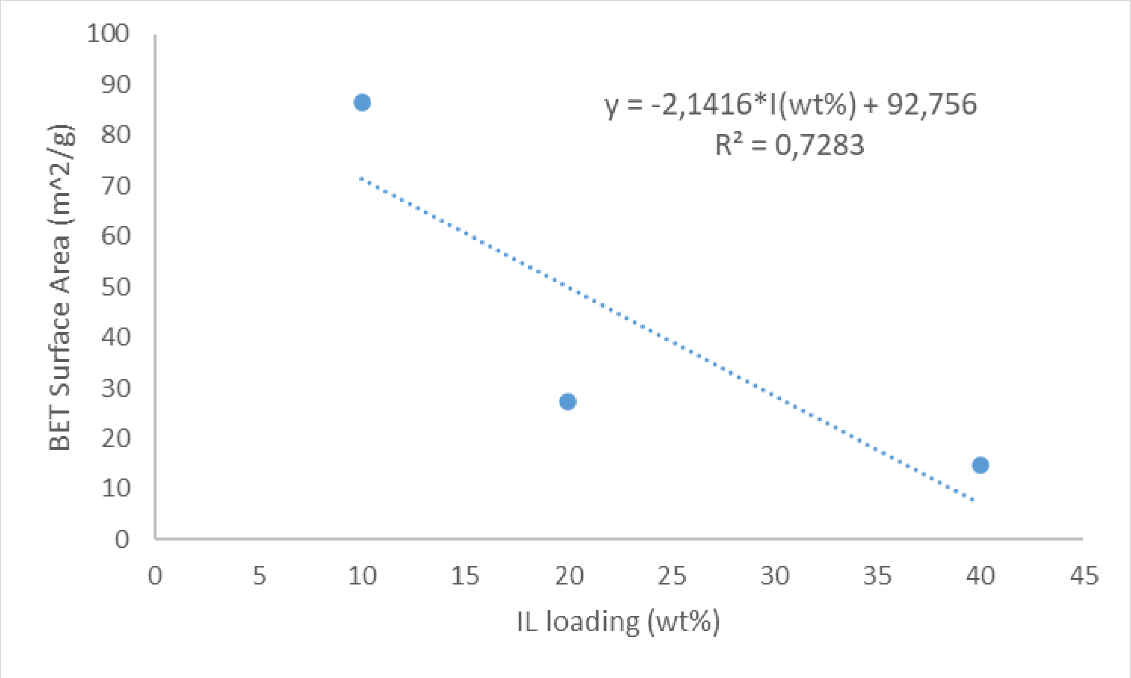

Thermal Gravimetric Analysis (TGA)

Figure S10. Thermogravimetric analysis of **[BMIM][PF$_6$]/CIM91 (40 wt%), [BMIM][PF$_6$]/CIM91 (20 wt%),** and **[BMIM][PF$_6$]/CIM91 (10 wt%).**

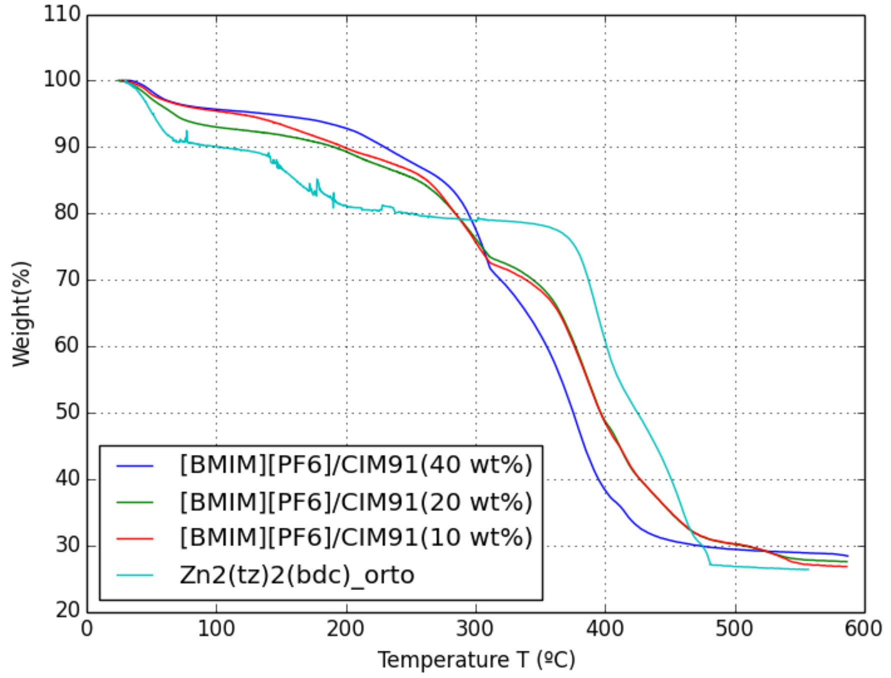

Figure S11. Thermogravimetric analysis of **[BMIM][Cl]/CIM91 (20 wt%), [BMIM][Cl]/CIM91 (10 wt%), [BMIM][Cl]/CIM91 (5 wt%) and CIM91.**

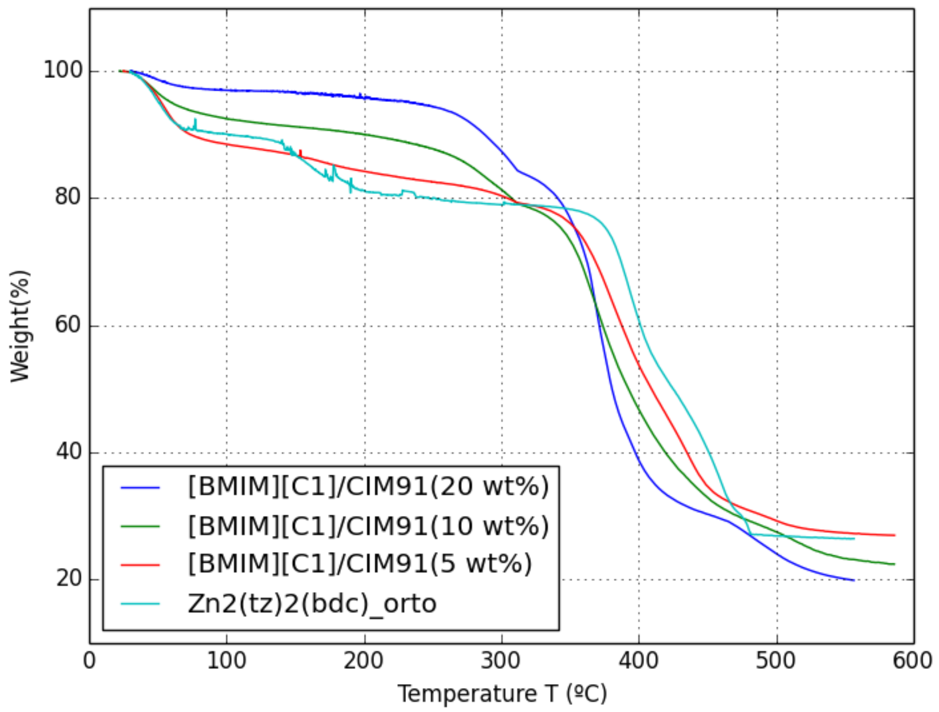

Infrared Analysis (IR)

Table S4. Characteristics peaks of [BMIM][Cl], CIM 91 and [BMIM][Cl]/CIM 91 (5, 10, 20% wt)

	**[BMIM][Cl]**	**CIM 91**	**[BMIM][Cl]/CIM 91 (5%)**	**[BMIM][Cl]/CIM91 (10%)**	**[BMIM][Cl]/CIM 91 (20%)**
**Stretching vibration of n-butyl hydrogen**	3443	-	3426	3442	3453
**Triazole ring CH symmetric stretching**	3096	-	3120	3111	-
**Aliphatic asymmetric and symmetric C-H stretching vibration**	2962,2875	-	-	2963, 2875	2961, 2874
**Stretching vibration of C=O**	-	-	1704	1702	-
**Asymmetric stretching vibration of carboxylate group**	-	1594	1587	1593	1597
**Symmetric stretching vibration of carboxylate group**	-	1388	1384	1367	1363
**(C=C) and C=N stretching vibration of triazole group**	-	1523	1522	1512	1509
**C-O vibration mode of Carboxylic group**	-	1297	1300	1296	1294
**C-N tension vibration of amine group**	-	1090	1092	1089	1084
**Triazole ring**	-	1004	1006	1006	1006
**In-plane C-H deformation vibration of triazole**	1169	-	1168	1169	1163
**C-N stretching**	844	-	840	833	830
**Ring bending**	623	-	623	623	622

Table S5. Characteristics peaks of CIM 91, $[BMIM][PF_6]$ and $[BMIM][PF_6]$/CIM 91 (10, 20, 40 % wt)

	**$[BMIM][PF_6]$**	**CIM91**	**$[BMIM][PF_6]$/CIM91 (10%)**	**$[BMIM][PF_6]$/CIM91 (20%)**	**$[BMIM][PF_6]$/CIM91 (40%)**
**Stretching vibration of n-butyl hydrogen**	3435	-	3448	3468	3449
**Triazole ring CH symmetric stretching**	3125	-	3138	3122	3138
**Aliphatic asymmetric and symmetric C-H stretching vibration**	2967,2940, 2879	-	2966, 2927, 2854	2966, 2876	2967
**Stretching vibration of C=O**	-	-	1701	1704	1701
**Asymmetric stretching vibration of carboxylate group**	-	1594	1586	1589	1581
**Symmetric stretching vibration of carboxylate group**	-	1388	1386	1386	1387
**(C=C) and C=N stretching vibration of triazole group**	-	1523	1522	1523	1522
**C-O vibration mode of Carboxylic group**	-	1297	1299	1300	1298
**C-N tension vibration of amine group**	-	1090	1093	1092	1092
**Triazole ring**	-	1006	1005	1006	1005
**Asymmetric vibration of $[PF_6]^-$**	842	-	843	842	845
**symmetric vibration of $[PF_6]^-$**	752	-	753	752	751
**In-plane C-H deformation vibration of triazole**	1169	-	1170	1170	1169
**Ring bending**	624	-	621	622	622

Figure S12. **.** FTIR spectroscopy of **CIM91, [BMIM][Cl], [BMIM][Cl]/CIM91 (5 wt%), [BMIM][Cl]/CIM91 (10 wt%), and [BMIM][Cl]/CIM91 (20 wt%).**

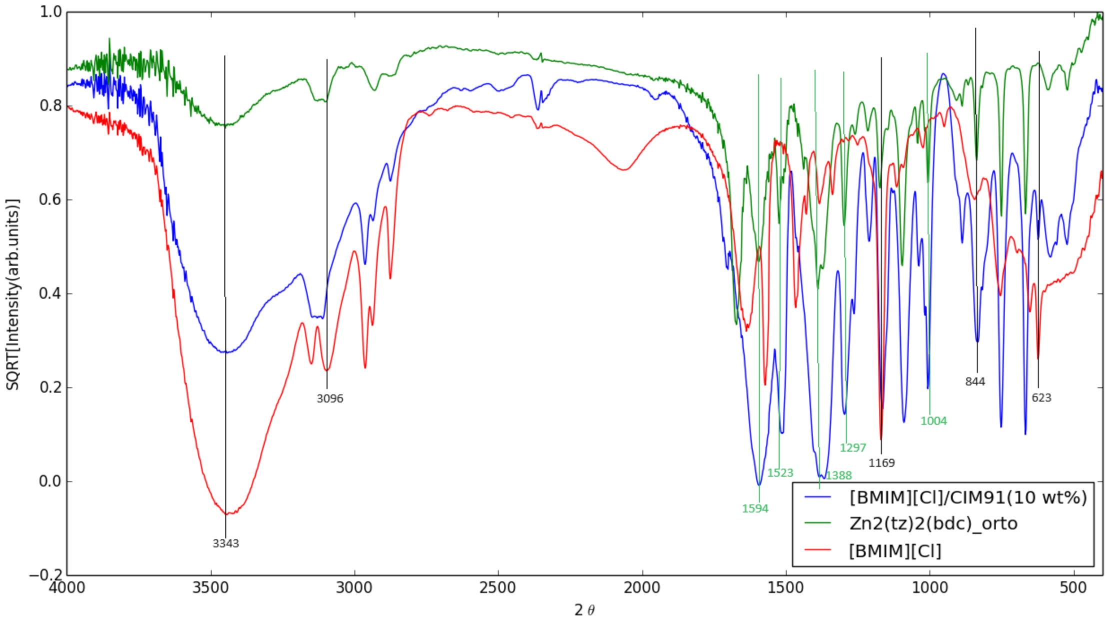

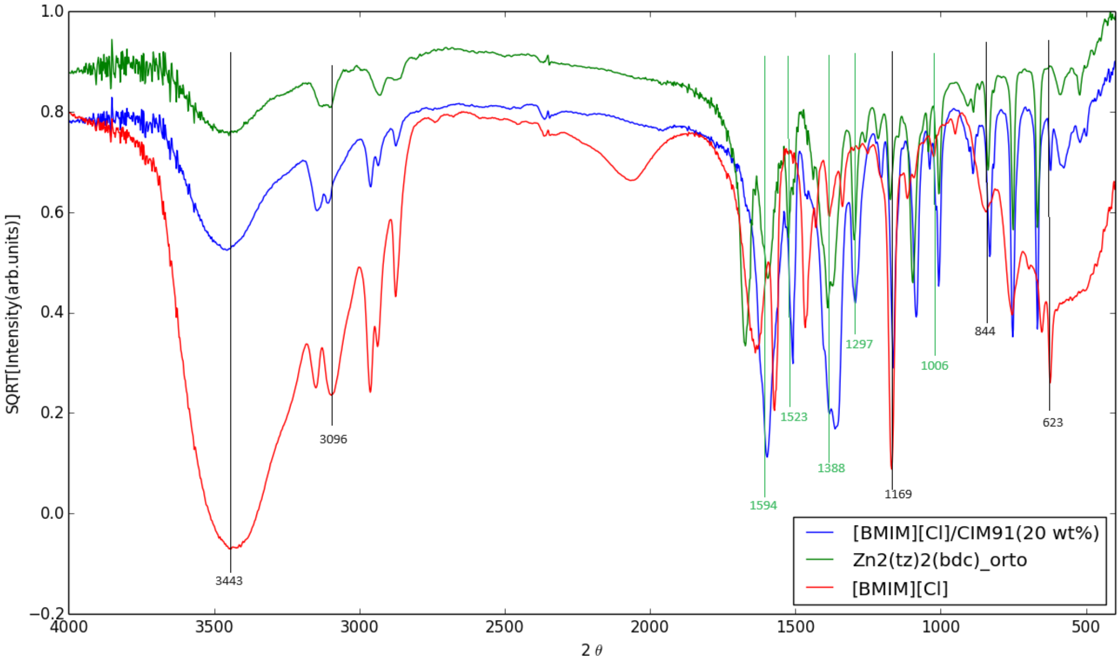

Figure S13. **.** FTIR spectroscopy of **CIM91, [BMIM][PF_6], [BMIM][PF_6]/CIM91 (10 wt%), [BMIM][PF_6]/CIM91 (20 wt%), and [BMIM][PF_6]/CIM91 (40 wt%).**

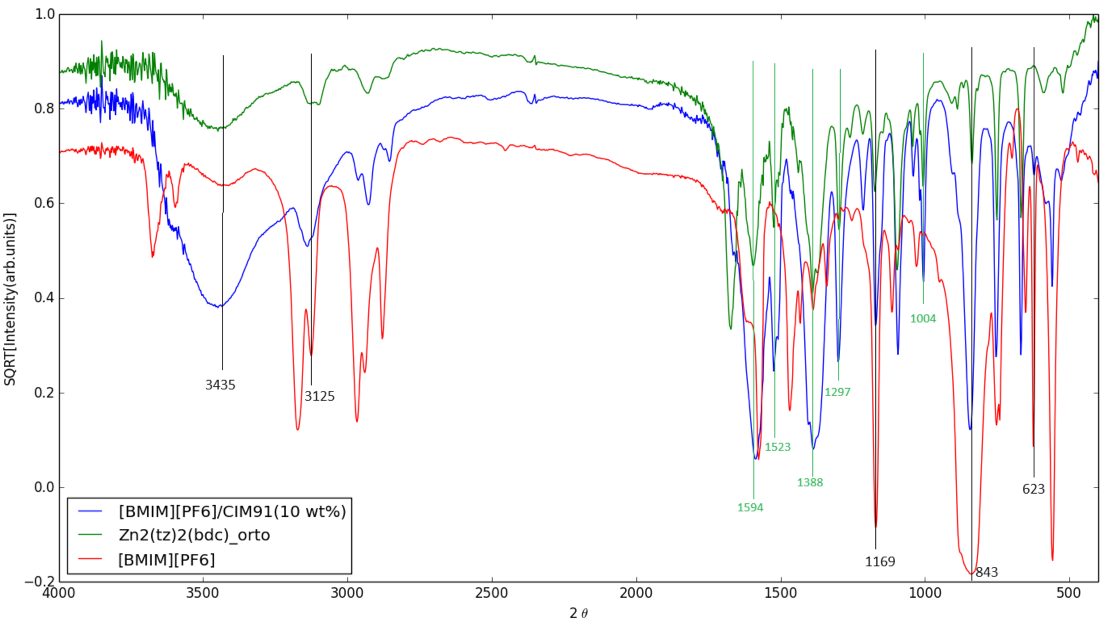
SQRT[Intensity(arb.units)]
2 θ
3435
3125
1594
1523
1388
1297
1169
1004
843
623
[BMIM][PF6]/CIM91(10 wt%)
Zn2(tz)2(bdc)_orto
[BMIM][PF6]

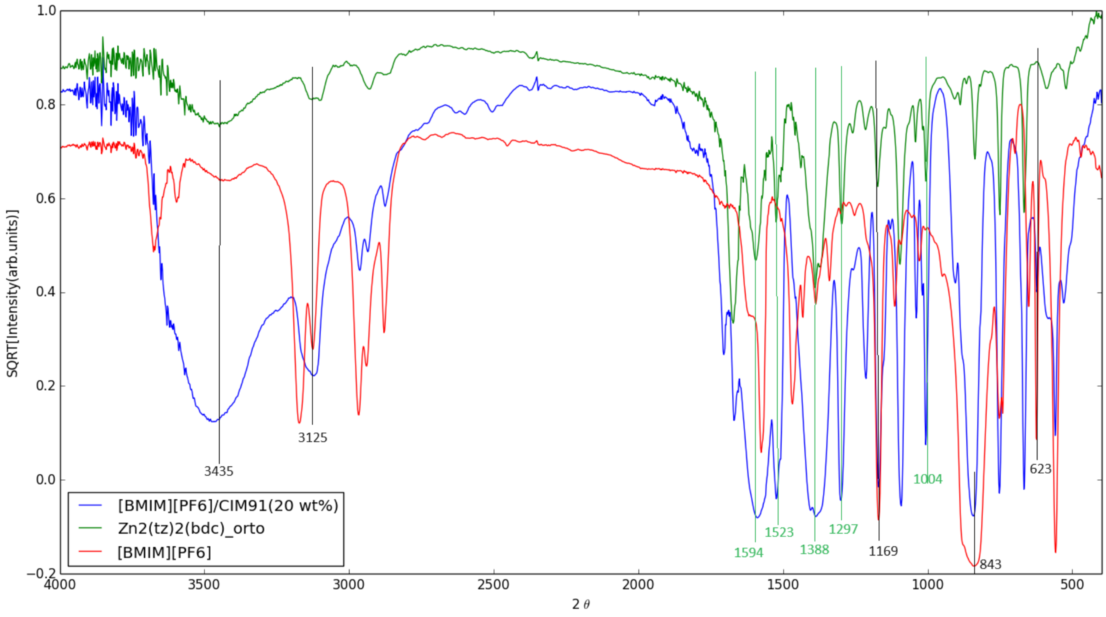
SQRT[Intensity(arb.units)]
2 θ
3435
3125
1594
1523
1388
1297
1169
1004
843
623
[BMIM][PF6]/CIM91(20 wt%)
Zn2(tz)2(bdc)_orto
[BMIM][PF6]

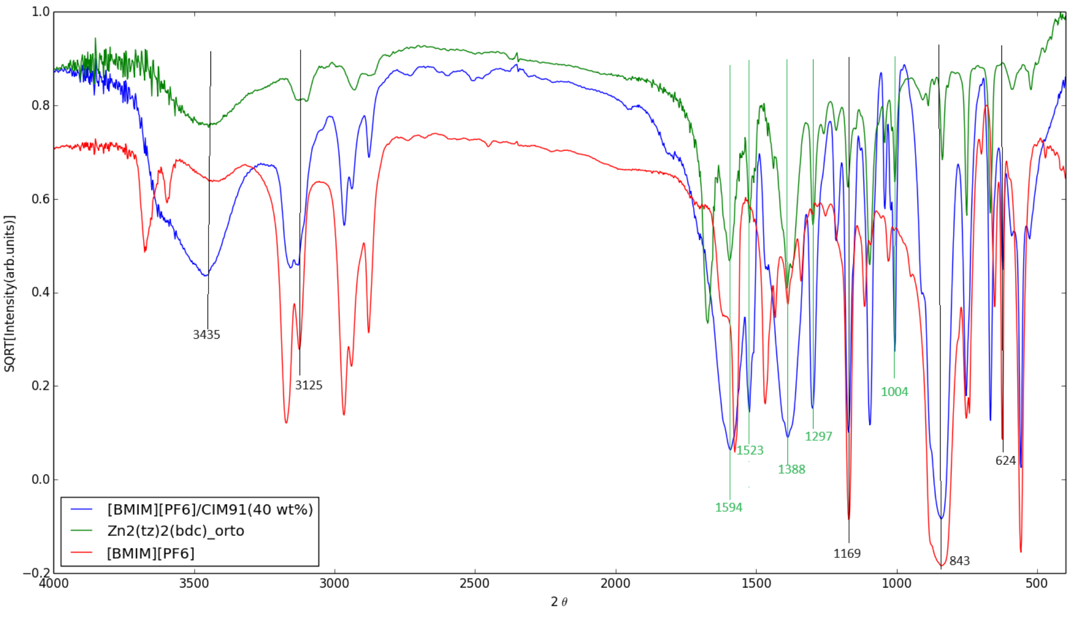
SQRT[Intensity(arb.units)]
2 θ
3435
3125
1594
1523
1388
1297
1169
1004
843
624
[BMIM][PF6]/CIM91(40 wt%)
Zn2(tz)2(bdc)_orto
[BMIM][PF6]

CO_2 Adsorption

Figure S14. Single-component adsorption-desorption equilibrium isotherms of CO_2 in the pristine CIM91 at a) 298 K (left) and b) 318 K (right). Represented: Vol ads/ g *versus* P(bar)

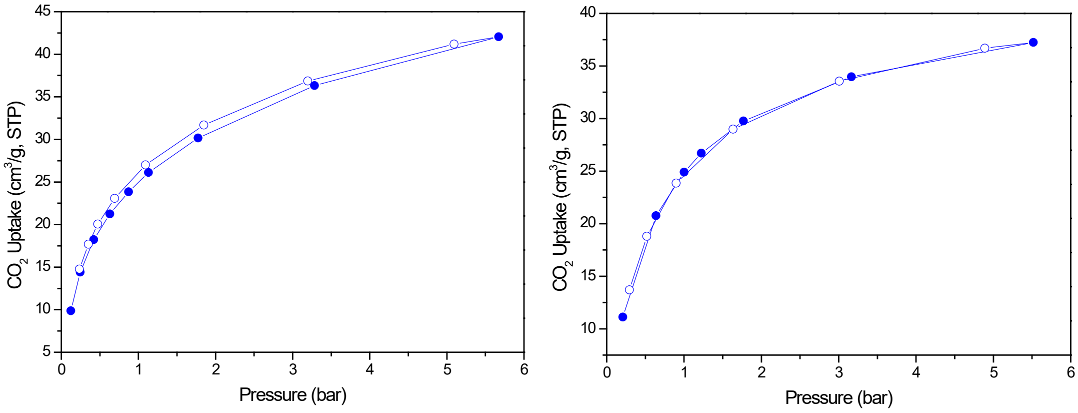

Figure S15. Single-component adsorption-desorption equilibrium isotherms of CO_2 in the **[BMIM][PF$_6$]/CIM91** samples at 298 K and 318 K. Represented: Vol ads/ g *versus* P(bar)

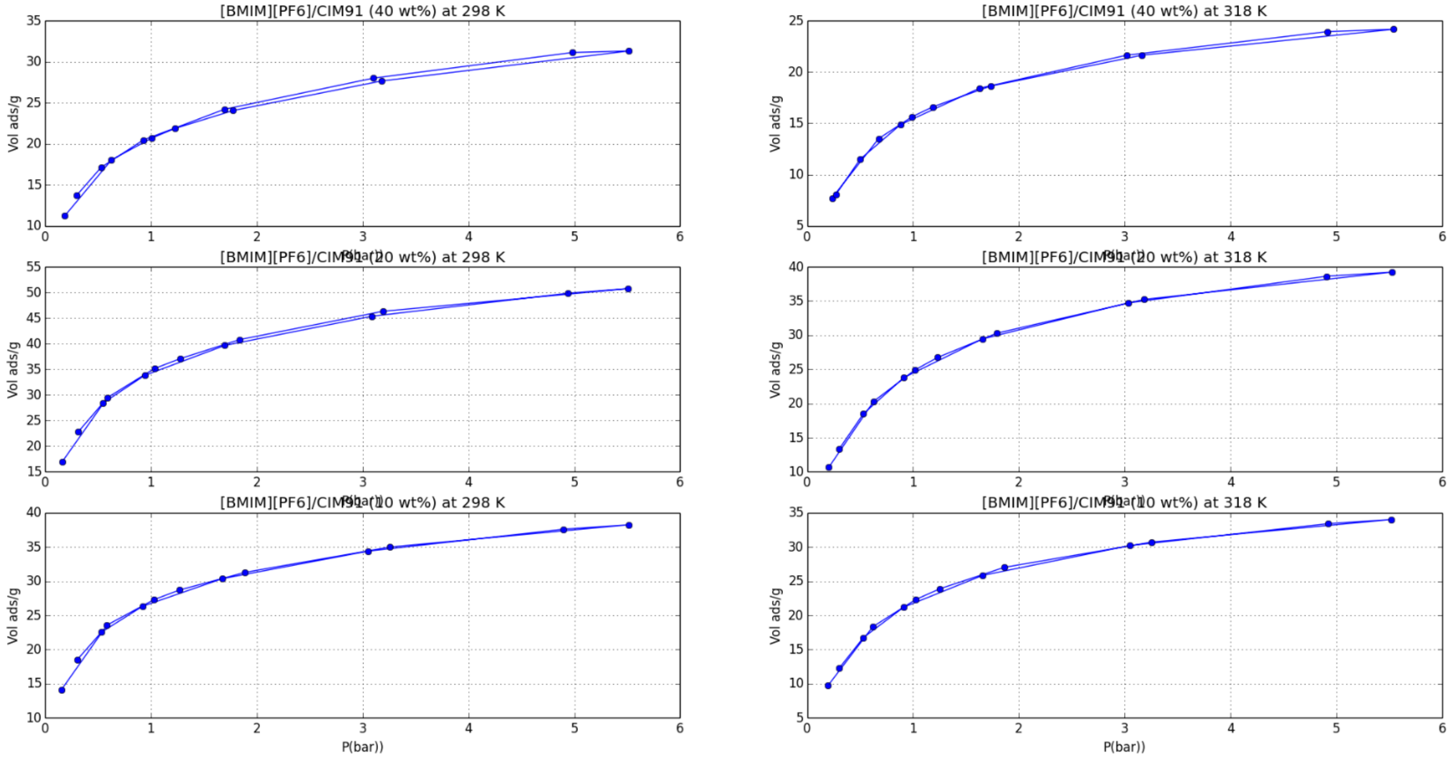

Figure S16. Single-component adsorption-desorption equilibrium isotherms of CO_2 in the **[BMIM][Cl]/CIM91** samples at 298 K and 318 K. Represented: Vol ads/ g *versus* P(bar)

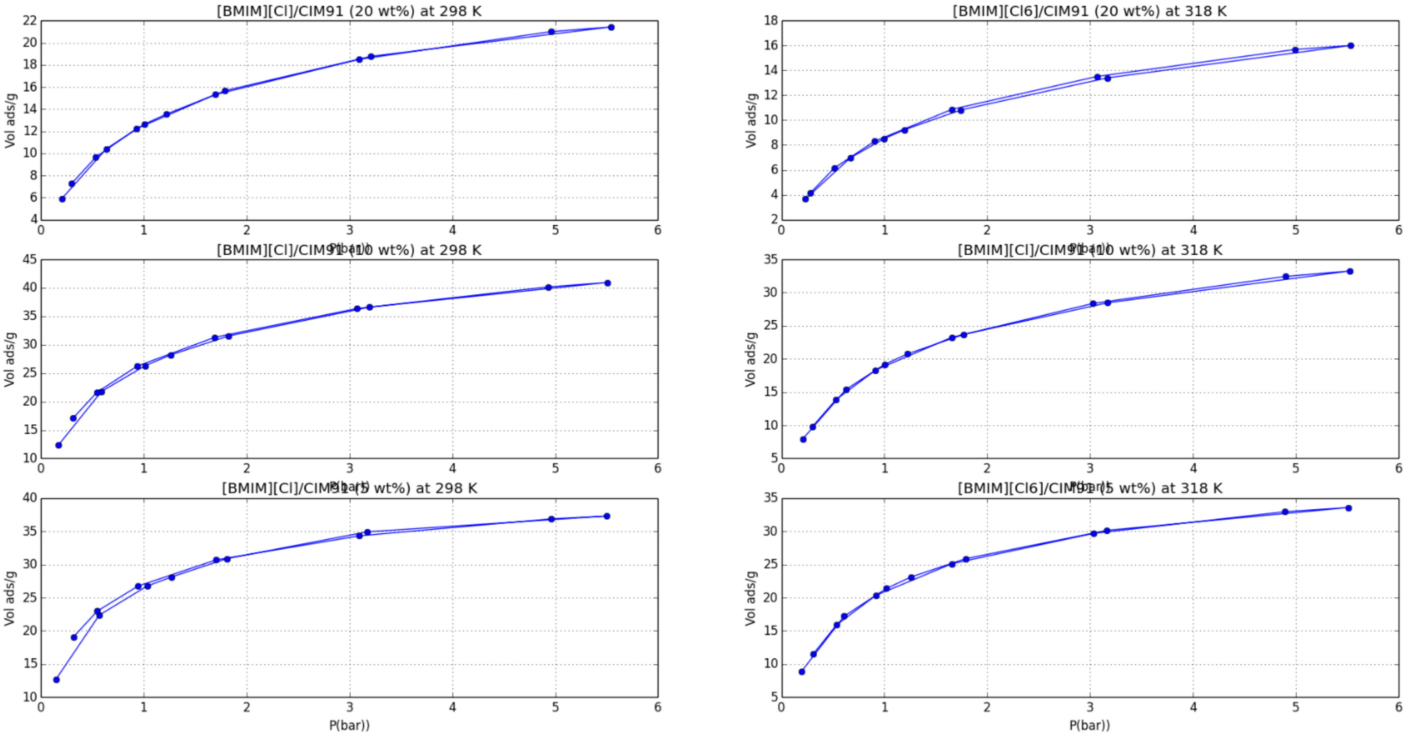

[BMIM][Cl]/CIM91 (20 wt%) at 298 K
[BMIM][Cl6]/CIM91 (20 wt%) at 318 K
[BMIM][Cl]/CIM91 (10 wt%) at 298 K
[BMIM][Cl]/CIM91 (10 wt%) at 318 K
[BMIM][Cl]/CIM91 (5 wt%) at 298 K
[BMIM][Cl6]/CIM91 (5 wt%) at 318 K
Vol ads/g
P(bar))

Once the CO_2 adsorption equilibrium points were accurately fitted, the heat of adsorption was estimated using the Clausius-Clapeyron equation.

$$Q_{st} = R \cdot \left(\frac{\partial \ln (P)}{\partial (1/T)}\right)_q \quad (1)$$

Figure S17. Heat of CO_2 adsorption estimated for all materials using Clausius-Clapeyron equation

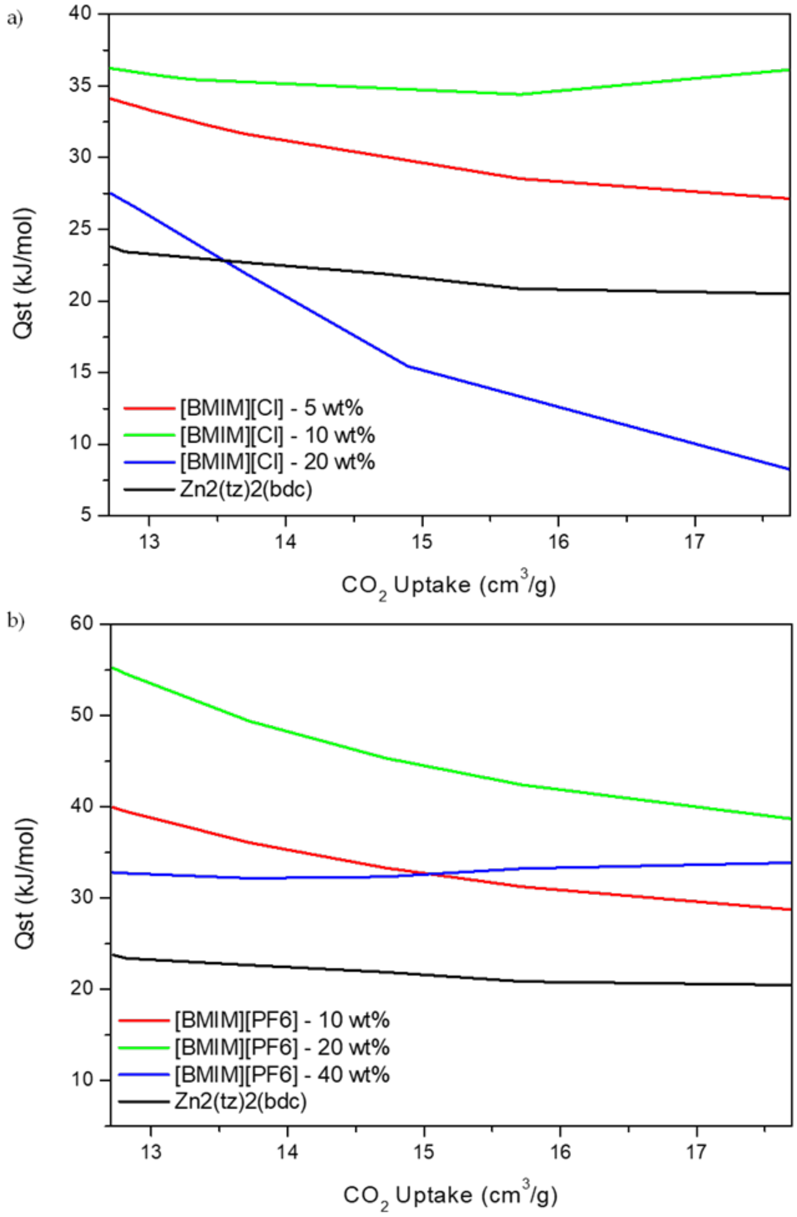

.

Dye Adsorption

The absorption study of methylene blue (MB) has been carried out both in water and in the case of [BMIM][PF_6] in ethanol. The dyes have been prepared in concentrations of 1, 5, 10 and 25 ppm (mg/L) in 20 mL of the solvent.

In a typical procedure, CIM91 and the different composites (20 mg) were added into MB solutions (20 mL, different concentrations). The changes in concentration for these samples were evaluated by UV–visible spectra.

Below, the variation in absorbance is presented as a function of the time ((0, 40, 60, 120, 180, 360, 900 and 1440 min). We performed the study for 24h in all samples The entire absorption spectrum is shown and a representation of the intensity of the normalized absorbance maximum as a function of time has also been done.

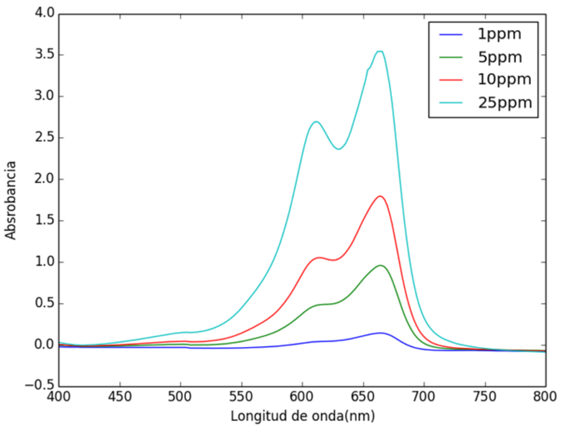

Figure S18. UV-visible spectra of MB at different concentrations in water

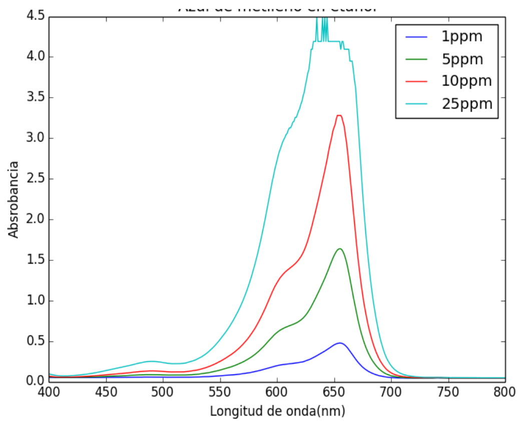

Figure S19. UV-visible spectra of different concentrations of MB in ethanol

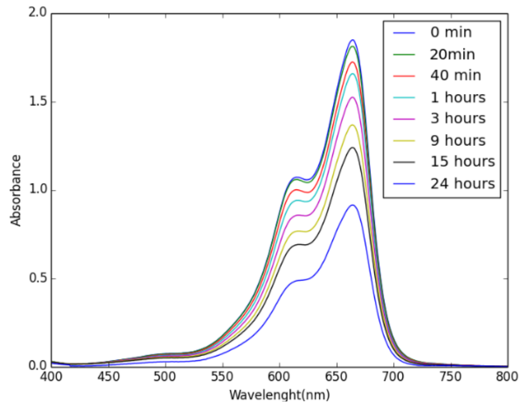

Figure S20 .UV-visible spectra of MB 10 ppm absorbed by CIM91 in water

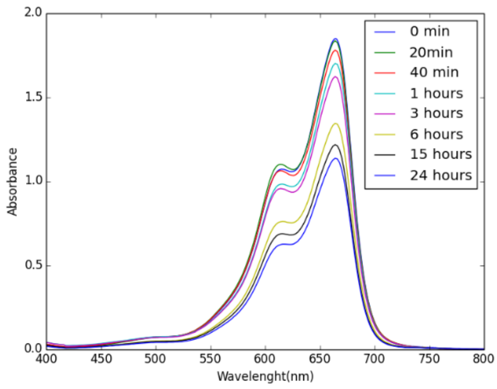

Figure S21. UV-visible spectra of MB 10 ppm absorbed by [BMIM][PF_6]/CIM91(10 wt%) in water

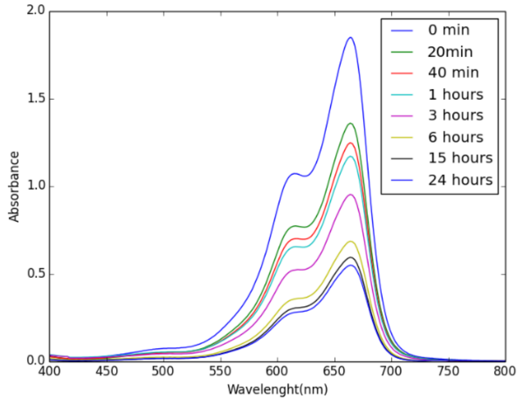

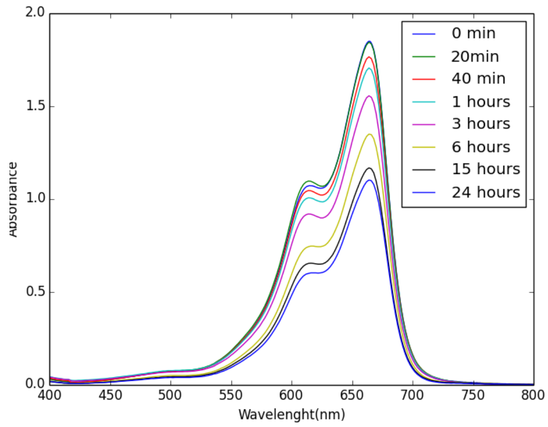

Figure S22. UV-visible spectra of MB 10 ppm absorbed by [BMIM][PF_6]/CIM91(20 wt%) in water

Figure S23. UV-visible spectra of MB 10 ppm absorbed by [MIM][PF_6]/CIM91(40 wt%) in water

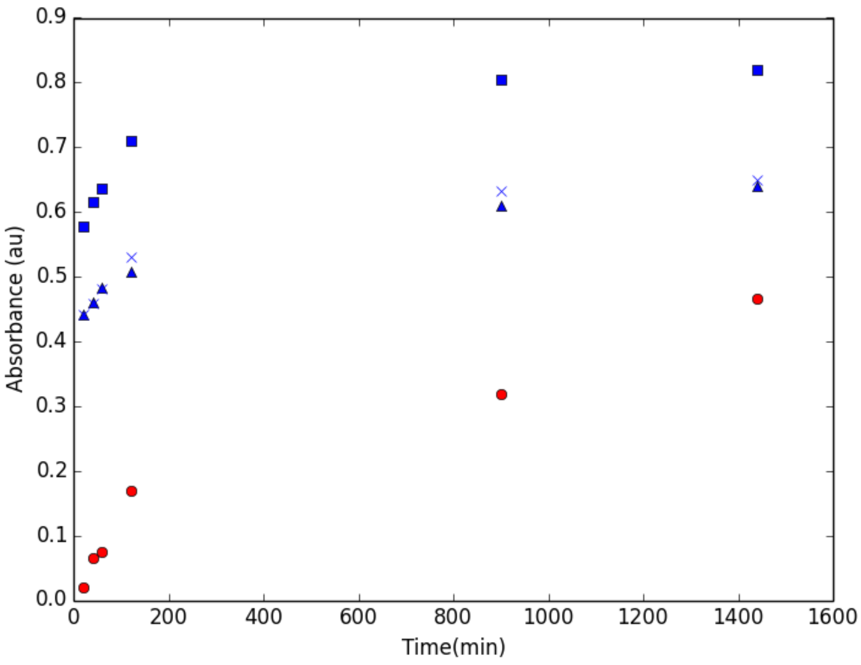

Figure S24. Maximum absorbance of pristine CIM91 (circle), [BMIM][PF_6]/CIM91 (10%) (triangle), [BMIM][PF_6]/CIM91 (20%) (square) and [BMIM][PF_6]/CIM91 (40%) (cross) in water over time.

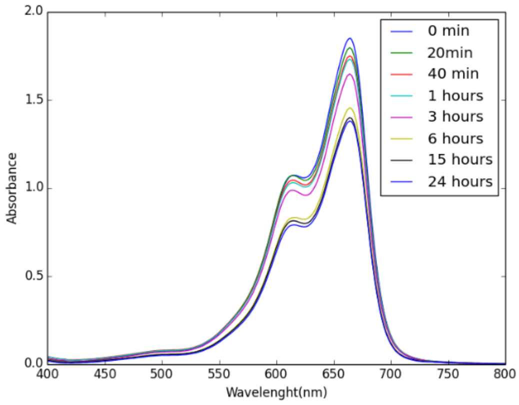

Figure S25. UV-visible spectra of MB 10 ppm absorbed by [BMIM][Cl]/CIM91(20 wt%) in water

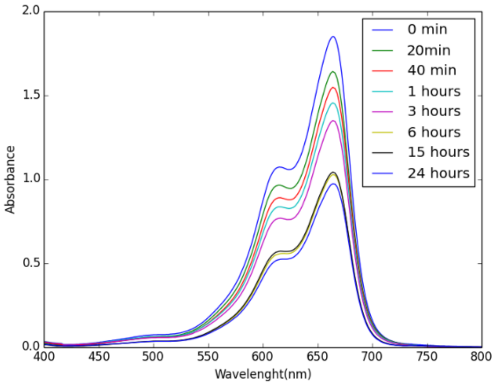

Figure S26. UV-visible spectra of MB 10 ppm absorbed by [BMIM][Cl]/CIM91(10 wt%) in water

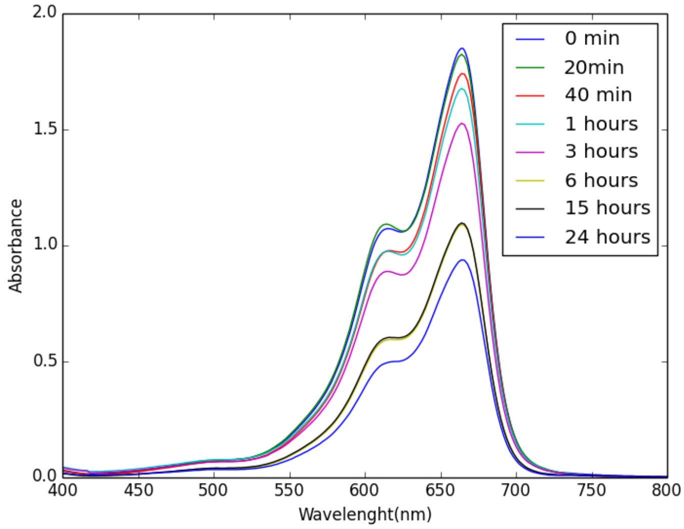

Figure S27. UV-visible spectra of MB 10 ppm absorbed by [BMIM][Cl]/CIM91(5 wt%) in water

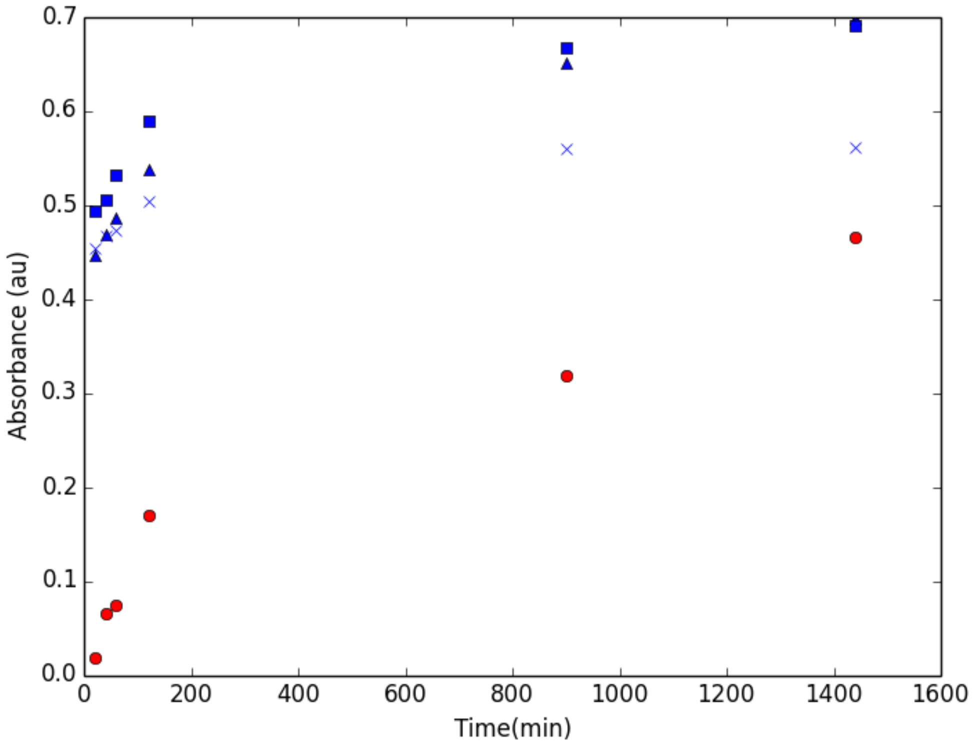

Figure S28. Maximum absorbance of pristine CIM91 (circle), [BMIM][Cl]/CIM91 (5%) (triangle), [BMIM][Cl]/CIM91 (10%) (square) and [BMIM][Cl]/CIM91 (20%) (cross) in water over time

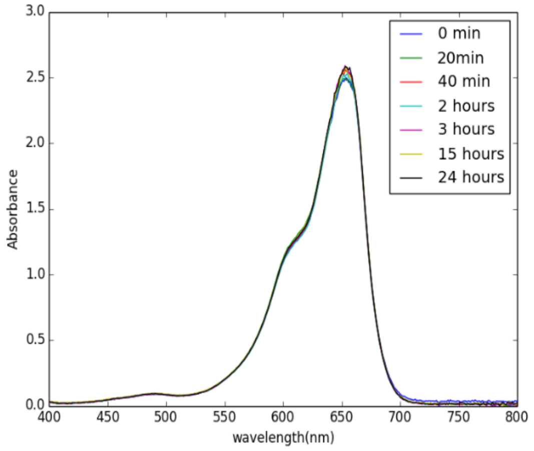

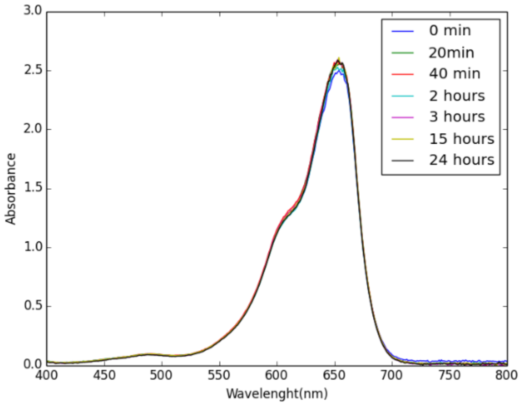

Figure S29. UV-visible spectra of MB 10 ppm absorbed by CIM91 in ethanol

Figure S30. UV-visible spectra of MB10 ppm absorbed by $[BMIM][PF_6]$/CIM91(10 wt%) in ethanol

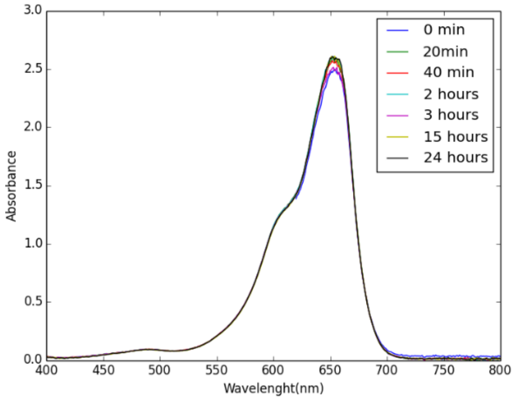

Figure S31. UV-visible spectra of MB 10 ppm absorbed by [BMIM][PF_6]/CIM91(20 wt%) in ethanol

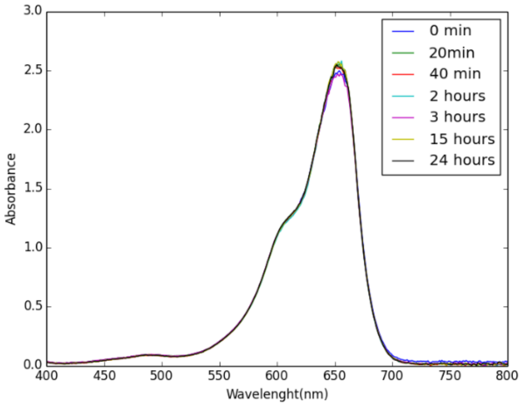

Figure S32. UV-visible spectra of MB 10 ppm absorbed by [BMIM][PF_6]/CIM91(40 wt%) in ethanol

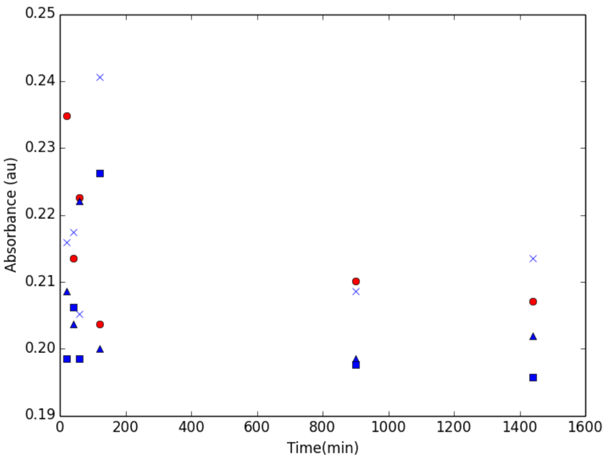

Figure S33. Maximum absorbance of pristine CIM91 (circle), [BMIM][PF_6]/CIM91 (10%) (triangle), [BMIM][PF_6]/CIM91 (20%) (square) and [BMIM][PF_6]/CIM91 (40%) (cross) in ethanol over time

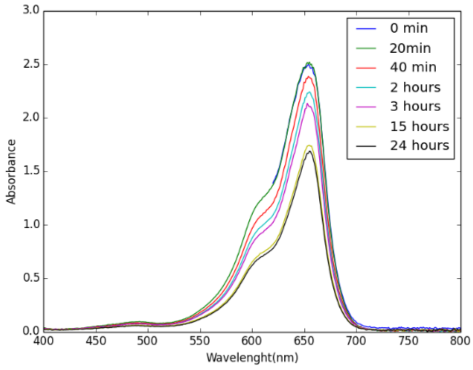

Figure S34. UV-visible spectra of MB 10 ppm absorbed by [BMIM][Cl]/CIM91(5 wt%) in ethanol

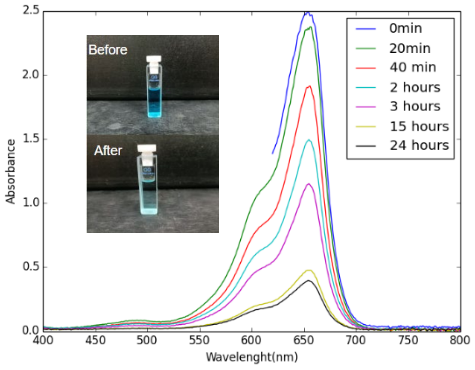

Figure S35. UV-visible spectra of MB 10 ppm absorbed by [BMIM][Cl]/CIM91(10 wt%) in ethanol

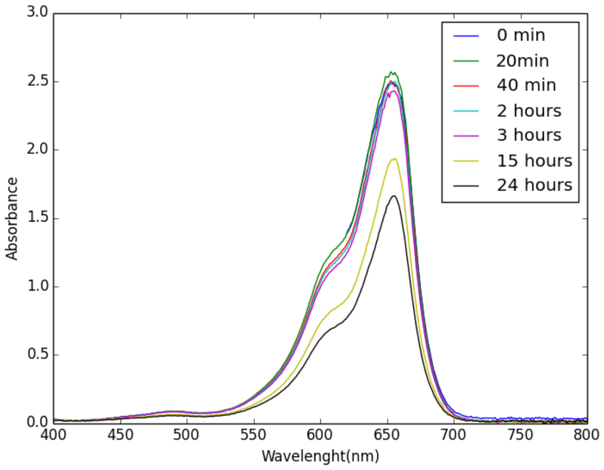

Figure S36. UV-visible spectra of MB 10 ppm absorbed by [BMIM][Cl]/CIM91(20 wt%) in ethanol

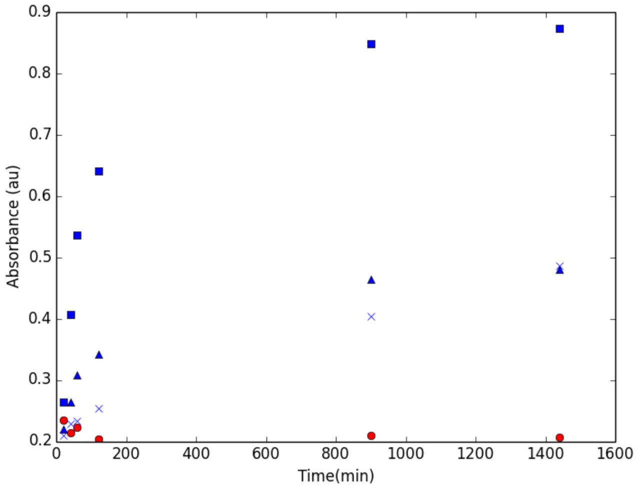

Figure S37. Maximum absorbance of pristine CIM91 (circle), [BMIM][Cl]/CIM91 (5%) (triangle), [BMIM][Cl]/CIM91 (10%) (square) and [BMIM][Cl]/CIM91 (20%) (cross) in ethanol over time

Tiempo 0 | Tiempo 15 horas | Tiempo 24 horas

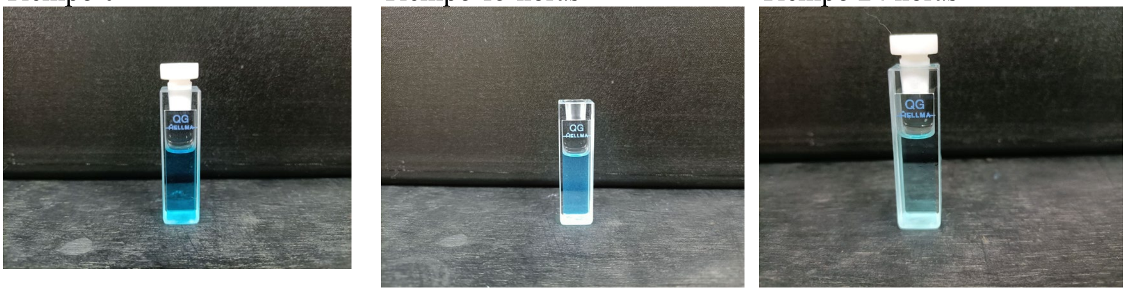

Figure S38. Methylene blue absorption by [BMIM][Cl](10%) over time

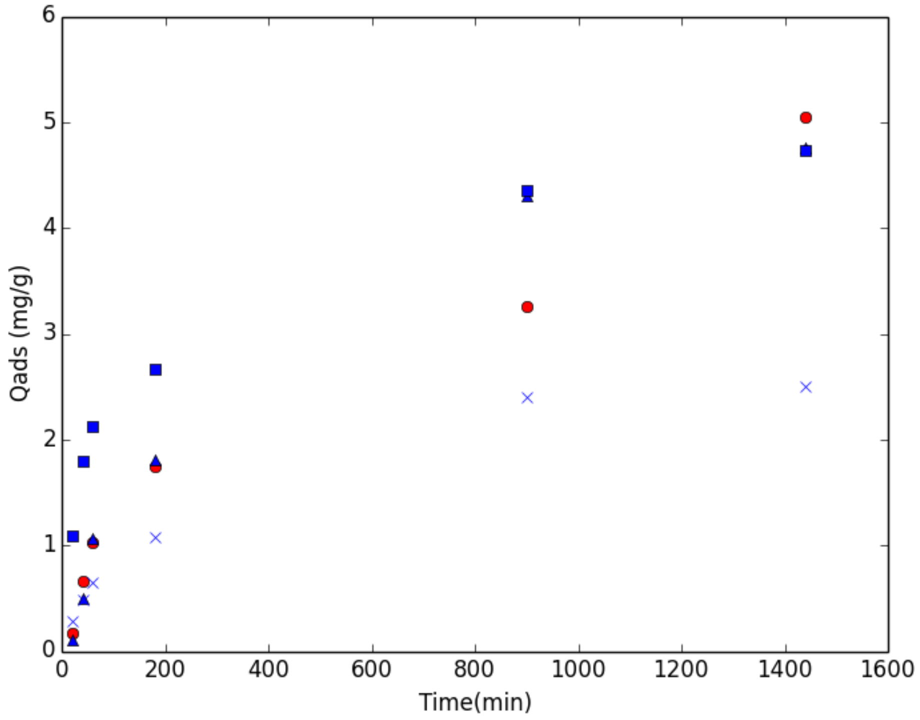

Figure S39. Time-dependent adsorption quantity of MB on CIM91 (circle), [BMIM][Cl]/CIM91 (5%) (triangle), [BMIM][Cl]/CIM91 (10%) (square) and [BMIM][Cl]/CIM91 (20%) (cross) in water

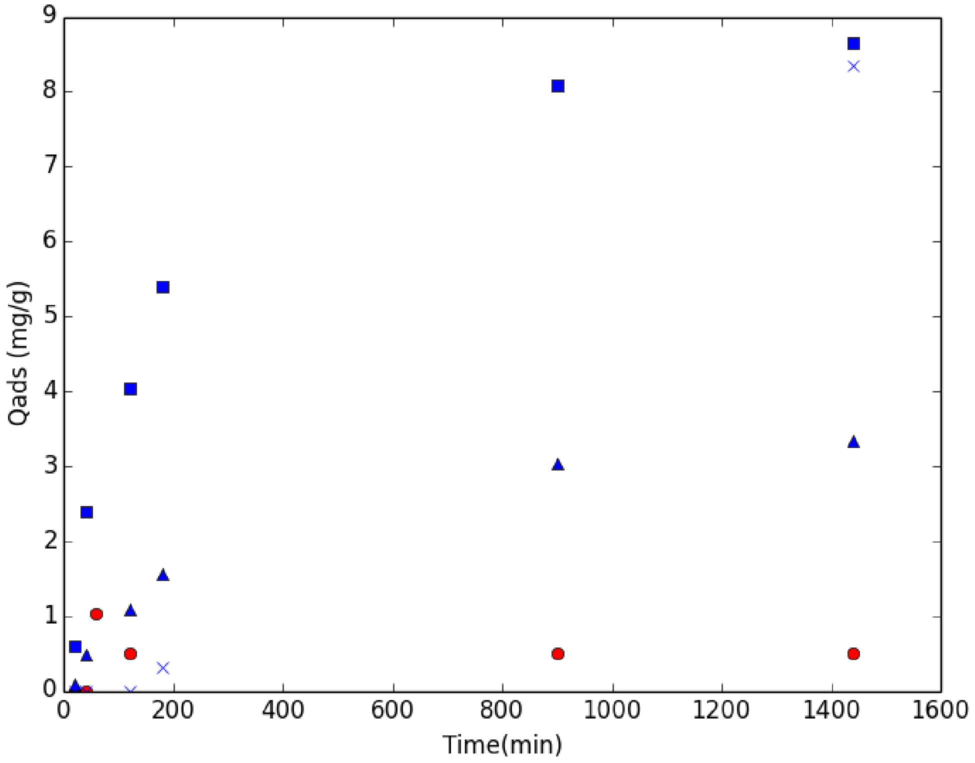

Figure S40. Time-dependent adsorption quantity of MB on CIM91 (circle), [BMIM][Cl]/CIM91 (5%) (triangle), [BMIM][Cl]/CIM91 (10%) (square) and [BMIM][Cl]/CIM91 (20%) (cross) in ethanol

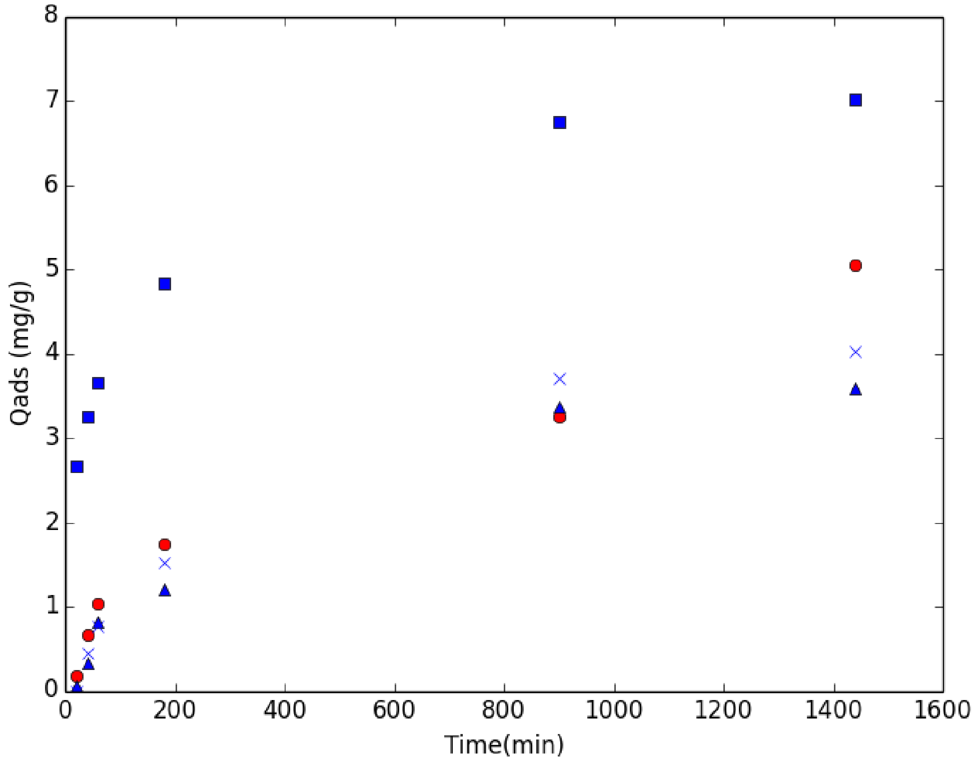

Figure S41. Time-dependent adsorption quantity of MB on CIM91 (circle), [BMIM][PF_6]/CIM91 (10%) (triangle), [BMIM][PF_6]/CIM91 (20%) (square) and [BMIM][PF_6]/CIM91 (40%) (cross) in water

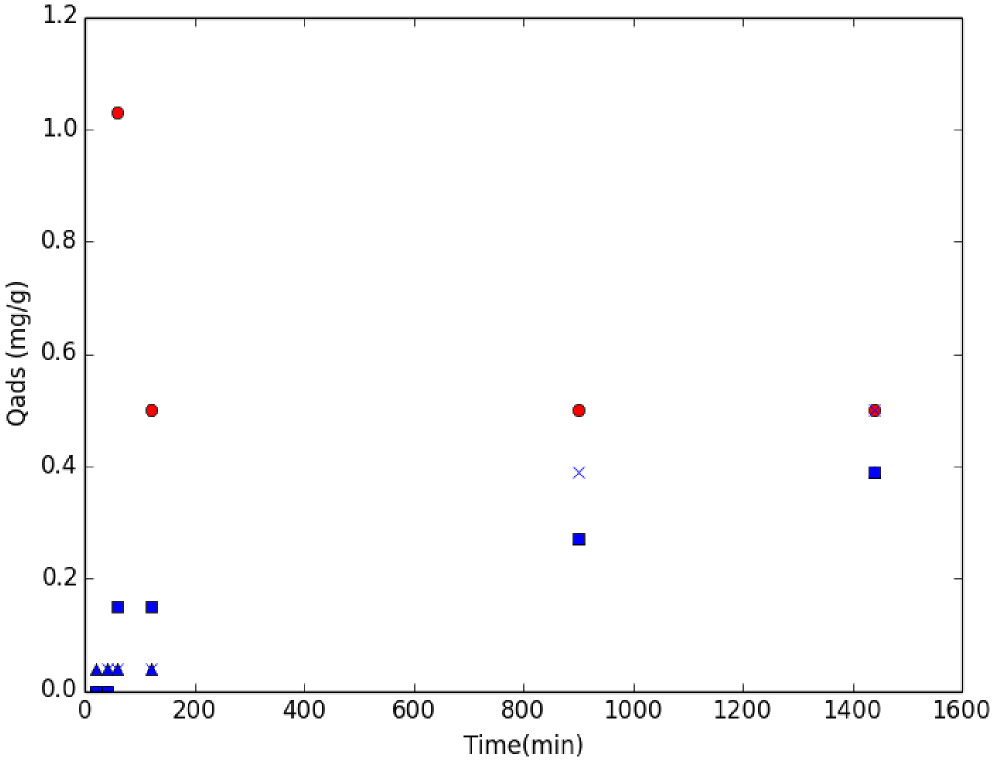

Figure S42. Time-dependent adsorption quantity of MB on CIM91 (circle), [BMIM][PF6]/CIM91 (10%) (triangle), [BMIM][PF6]/CIM91 (20%) (square) and [BMIM][PF6]/CIM91 (40%) (cross) in ethanol

The quantity and efficiency of MB could be calculated by the equations $Q = (C_0 - C_t)V/m$ and efficiency (%) = $(C_0 - C_t) \times 100/C_0$, respectively, where C_0 and C_t represent the concentration of the dye at the beginning and at a certain time (mg L^{-1}) and m and V are the adsorbent quality (g) and the dye volume (L), respectively.

Table S6. Quantity value and efficiency of MB for [BMIM][Cl]/CIM91 composite in ethanol and CIM 91 and [BMIM][PF_6]/CIM91 composite in water

Compound	t (min)	Q_t (mgL^{-1})	Q (mg/g)	Efficiency (%)
[BMIM][Cl]/CIM91 (5%) in ethanol	0	10	0	0
	20	9,92	0,08	0,8
	40	9,52	0,48	4,8
	120	8,92	1,08	10,8
	180	8,44	1,56	15,6
	900	6,96	3,04	30,4
	1440	6,67	3,33	33,3
[BMIM][Cl]/CIM91 (10%) in ethanol	0	10	0	0
	20	9,4	0,6	6
	40	7,6	2,4	24
	120	5,96	4,04	40,4
	180	4,6	5,4	54
	900	1,92	8,08	80,8
	1440	1,35	8,65	86,5
[BMIM][Cl]/CIM91 (20%) in ethanol	0	10	0	0
	20	10	0	0
	40	10	0	0

	120	10	0	0
	180	9,68	0,32	3,2
	900	1,92	8,08	80,8
	1440	1,66	8,34	83,4
CIM 91 in ethanol	0	10	0	0
	20	10	0	0
	40	9,5	0,5	5
	120	9,5	0,5	5
	180	9,5	0,5	5
	900	9,5	0,5	5
	1440	9,5	0,5	5
CIM 91 in water	0	10	0	0
	20	9,83	0,17	1,7
	40	9,34	0,66	6,6
	60	8,97	1,03	10,3
	180	8,26	1,74	17,4
	540	7,39	2,61	26,1
	900	6,74	3,26	32,6
	1440	4,95	5,05	50,5
[BMIM][PF6]/CIM91 (10 %) in water	0	10	0	0
	20	9,94	0,06	0,6
	40	9,67	0,33	3,3

	60	9,18	0,82	8,2
	180	8,8	1,2	12
	360	7,28	2,72	27,2
	900	6,63	3,37	33,7
	1440	6,41	3,59	35,9
[BMIM][PF6]/CIM91 (20 %) in water	0	10	0	0
	20	7,33	2,67	26,7
	40	6,74	3,26	32,6
	60	6,35	3,65	36,5
	180	5,16	4,84	48,4
	360	3,69	6,31	63,1
	900	3,26	6,74	67,4
	1440	2,99	7,01	70,1
[BMIM][PF6]/CIM91 (40 %) in water	0	10	0	0
	20	10	0	0
	40	9,56	0,44	4,4
	60	9,23	0,77	7,7
	180	8,48	1,52	15,2
	360	7,33	2,67	26,7
	900	6,3	3,7	37
	1440	5,98	4,02	40,2
